# Transcriptomic signatures of the insular cortex in a mouse model of neuropathic pain

**DOI:** 10.3389/fnmol.2026.1840950

**Published:** 2026-07-02

**Authors:** Yang Bai, Guo-Quan Yao, Cheng-Guo Jiang, Chong Zhang, Yu-Gang Diao, Si-Zhe Feng, Guo-Biao Liang, Xin-Tong Qiu, Wei Jiang

**Affiliations:** 1 Department of Neurosurgery, General Hospital of Northern Theater Command, Shenyang, China; 2 Department of Anesthesiology, General Hospital of Northern Theater Command, Shenyang, China; 3 Key Laboratory of Perioperative Critical Medicine of Liaoning Province, Shenyang, China; 4 Shenyang Clinical Medical Research Center for Anesthesiology and Perioperative Medicine, Shenyang, China; 5 Department of Anatomy, Histology and Embryology, Preclinical School of Medicine, Air Force Medical University, Xi’an, China

**Keywords:** insular cortex, neurodegenerative disease, neuropathic pain, synaptic plasticity, transcriptomics

## Abstract

**Background:**

Neuropathic pain (NP) remains poorly managed by current therapies. Although the insular cortex (IC) is critical for cortical pain processing, a comprehensive spatiotemporal molecular characterization of the IC in NP is lacking.

**Methods:**

We employed RNA sequencing of the anterior (aIC) and posterior (pIC) insular cortices at 2 and 4 weeks following spared nerve injury in mice. Integrative bioinformatics analyses—including differential expression, functional enrichment, weighted gene co-expression network analysis, and protein–protein interaction (PPI) network construction—were used to delineate the molecular landscape.

**Results:**

Widespread transcriptional dysregulation in the IC was observed, with the number of differentially expressed genes increasing over time. Functional analyses reaffirmed involvement of neuroinflammation and synaptic plasticity-related pathways and further identified dysregulation of mitochondrial pathways—mechanisms commonly implicated in neurodegenerative disorders. Subregion analysis revealed that the aIC exhibited broader and more persistent pathway alterations than the pIC, including programmed cell death (early phase), mitochondrial dysfunction/neurodegeneration (late phase), indicating a progressive stress response unique to the aIC. PPI network analysis identified stage-specific hub genes: early-phase interferon-stimulated genes predominated in both subregions; late-phase hub genes included circadian rhythm regulators, ER stress markers and inflammatory mediators.

**Conclusion:**

This study presents a detailed transcriptomic profile of the IC in NP, revealing region- and time-dependent remodeling. Our results confirm known mechanisms and uncover dysregulation reminiscent of neurodegenerative disorders—predominantly in the aIC, suggesting its heightened susceptibility to pain-induced pathology. These findings expand our understanding of IC-mediated pathophysiological processes in NP and may provide a framework for identifying novel therapeutic targets for chronic pain.

## 1 Introduction

Neuropathic pain (NP), a debilitating condition resulting from injury or dysfunction of the somatosensory system, affects approximately 10% of the global population (Finnerup et al., 2021). This condition imposes a substantial burden on patients and remains a major clinical challenge in pain medicine (Scholz et al., 2019). Current pharmacotherapies—primarily central nervous system (CNS)-targeted agents such as opioids and antidepressants—offer limited efficacy, and are often complicated by safety concerns, leaving clinical needs largely unmet (Cohen et al., 2021). This therapeutic gap underscores the necessity of advancing our understanding of the molecular underpinnings of NP. Profiling gene and protein expression in pain-related neural circuits is crucial for deciphering NP pathophysiology and informing the development of novel, mechanism-based treatments (Starobova et al., 2018; Gomez-Varela et al., 2019).

The insular cortex (IC), a central hub of the limbic system, integrates multimodal salient information—spanning sensory inputs to cognitive-affective processes—to underpin conscious interoception (Gogolla, 2017; Zhang R. et al., 2024). In pain processing, the IC contributes to both the sensory-discriminative and affective-motivational dimensions of pain perception. Anatomical subdivisions defined by the central insular sulcus distinguish the anterior IC (aIC) from the posterior IC (pIC). Anatomical (Lu et al., 2016; Gehrlach et al., 2020) and neuroimaging (Schreckenberger et al., 2005; Peltz et al., 2011) studies suggest that the aIC is preferentially involved in the affective dimension of pain, whereas the pIC is more closely linked to its sensory-discriminative component, consistent with their respective preferential connectivity with limbic-cognitive and sensory-processing regions, respectively. Behavioral evidence corroborates this functional dissociation (Barthas et al., 2015; Bai et al., 2019; Salimi and Tamaddonfard, 2019), motivating recent efforts using advanced techniques (including optogenetics) to uncover circuit-specific mechanisms through which the IC modulates pain (Tan et al., 2017; Lee et al., 2022; Meng et al., 2022; Zhang M. et al., 2022; Zhang F. et al., 2024). Meanwhile, integrated molecular and pharmacological approaches are revealing the neurochemical mechanisms underlying pain-related changes in the IC (Lu et al., 2016; Wang et al., 2021). Despite this evidence, remarkably less is known about the molecular basis underlying IC neurobiology in NP.

The past decade has seen a fundamental transformation in pain research, driven by the emergence of multi-omics biology. This paradigm enables a shift from traditional qualitative studies focused on single targets toward systematic exploration of multidimensional cellular networks. High-throughput technologies such as RNA sequencing now enable large-scale profiling of pain-related molecules (Nie et al., 2007; Niederberger and Geisslinger, 2008). In pain research, mRNA expression analyses have been widely applied across different levels of the nervous system—from primary sensory neurons and the spinal dorsal horn (SDH) (Niederberger and Geisslinger, 2008; Starobova et al., 2018) to subcortical and cortical regions (Su et al., 2021; Dai et al., 2022; Zhang Y. et al., 2022). Nevertheless, the comprehensive transcriptomic landscape of the IC in NP remains largely unexplored. Here, we characterize the temporal transcriptomic dynamics of NP in the functionally distinct anterior and posterior subregions of the IC. Using longitudinal RNA-seq profiling at 14 and 28 days post-spared nerve injury (SNI), we delineate subregion-specific and time-dependent molecular reprogramming associated with the persistence of chronic pain. This advances our understanding of the complexity of cortical pain processing and regulation and offers a new framework for identifying precise therapeutic opportunities.

## 2 Materials and methods

### 2.1 Animals

All procedures involving animals were reviewed and approved by the Animal Ethics Committee of the General Hospital of Northern Theater Command and were conducted in strict compliance with the National Institutes of Health Guide for the Care and Use of Laboratory Animals. A total of 85 adult male C57BL/6 mice (8 weeks old; 20–30 g) were obtained from the experimental animal center of the hospital. Animals were housed under a 12-h reverse light/dark cycle with free access to standard rodent diet and water. The animal study protocol was approved by the Institutional Review Board of the General Hospital of Northern Theatre Command (protocol code 2025-MSLH-731).

### 2.2 Neuropathic pain model induction and behavioral validation

All surgical procedures were performed as previously described (Ma et al., 2022). Briefly, mice were placed under deep anesthesia, and a longitudinal incision was made in the skin of the left hind limb to expose the biceps femoris muscle. The sciatic nerve and its three terminal branches—the sural, common peroneal, and tibial nerves—were then dissected. Both the common peroneal and tibial nerves were ligated using 6-0 silk suture and transected distal to the ligation site, while the sural nerve was left intact. The muscle and skin layers were subsequently closed with sutures. Sham-operated animals underwent identical surgical exposure of the sciatic nerve without nerve ligation or transection.

Mechanical withdrawal thresholds of the ipsilateral hindpaw were evaluated using von Frey filaments. Prior to behavioral testing, mice were habituated to handling and the testing environment through daily 30-min sessions over 3 consecutive days. During testing, a graded series of calibrated von Frey filaments were applied perpendicularly to the plantar surface of the hindpaw. Each filament was applied five times, with each application maintained for 5–8 s and an inter-stimulus interval of 5 min. The minimal filament force eliciting at least three distinct withdrawal responses out of five consecutive trials was recorded as the paw withdrawal threshold (PWT). Positive withdrawal responses were defined as rapid paw retraction, licking, shaking of the paw, or audible vocalization.

For open field tests, mice were first placed in the testing room for three consecutive days for acclimatization before testing. All behavioral tests were conducted between 9:00 a.m. and 2:00 p.m. During the experiments, dim red lighting was used and the room remained quiet. Thirty minutes prior to testing, animals were moved to the open field testing room for habituation. Subsequently, each mouse was placed in the center of a white-bottomed open field apparatus (50 cm × 50 cm × 45 cm). A video recording system (Shanghai Yishu Digital Technology Co., Ltd.) recorded mouse activity within the open field for 15 min. Following video capture, behavioral trajectories were analyzed using corresponding software. Total distance traveled in the open field was used as an indicator of locomotor ability, and the percentage of time spent in the central area (center time %) was used as an index of anxiety-like behavior.

For the behavioral data, the continuous variables were expressed as the mean ± standard deviation. Means were compared by One-way ANOVA followed by Turkey’s multiple comparisons test. Statistical analysis was conducted using the SPSS statistical software (IBM Corp., version: 26.0, NY, United States). A two-tailed *p* < 0.05 was considered significant.

### 2.3 Study design and tissue collection

Baseline mechanical sensitivity of the left hindpaw was assessed in all animals prior to SNI surgery using von Frey filaments. Mice exhibiting normal withdrawal thresholds (0.6, 1.0, or 1.4 g) were included, while four animals with abnormal thresholds ( < 0.6 g or > 1.4 g) were excluded. The remaining 81 mice were randomly assigned to three groups: sham, SNI-2w, and SNI-4w. The SNI procedure was performed on the left side to induce neuropathic pain. Mechanical thresholds of the ipsilateral plantar surface were reassessed at 2 and 4 weeks post-surgery to confirm successful model establishment. Of the 54 SNI-operated animals, 52 met the success criterion (withdrawal threshold ≤ 0.04 g), yielding a model success rate of 96.3%. One day after the final behavioral test, mice were euthanized by rapid cervical dislocation. From each group, 24 animals were selected for insular cortex collection.

To ensure anatomical precision, 300-μm-thick coronal brain sections containing the bilateral insular cortex were prepared using a vibratome in ice-cold, oxygenated artificial cerebrospinal fluid of the following composition (in mM): 2.6 KCl, 124 NaCl, 1 MgCl_2_, 0.5 CaCl_2_, 1.23 NaH_2_PO_2_, 26.2 NaHCO_2_, 5 kynurenic acid, 212.7 sucrose, 10 dextrose. The target insular cortical areas were then micro-dissected using a 15-gauge needle and immediately snap-frozen on dry ice.

**FIGURE 1:**
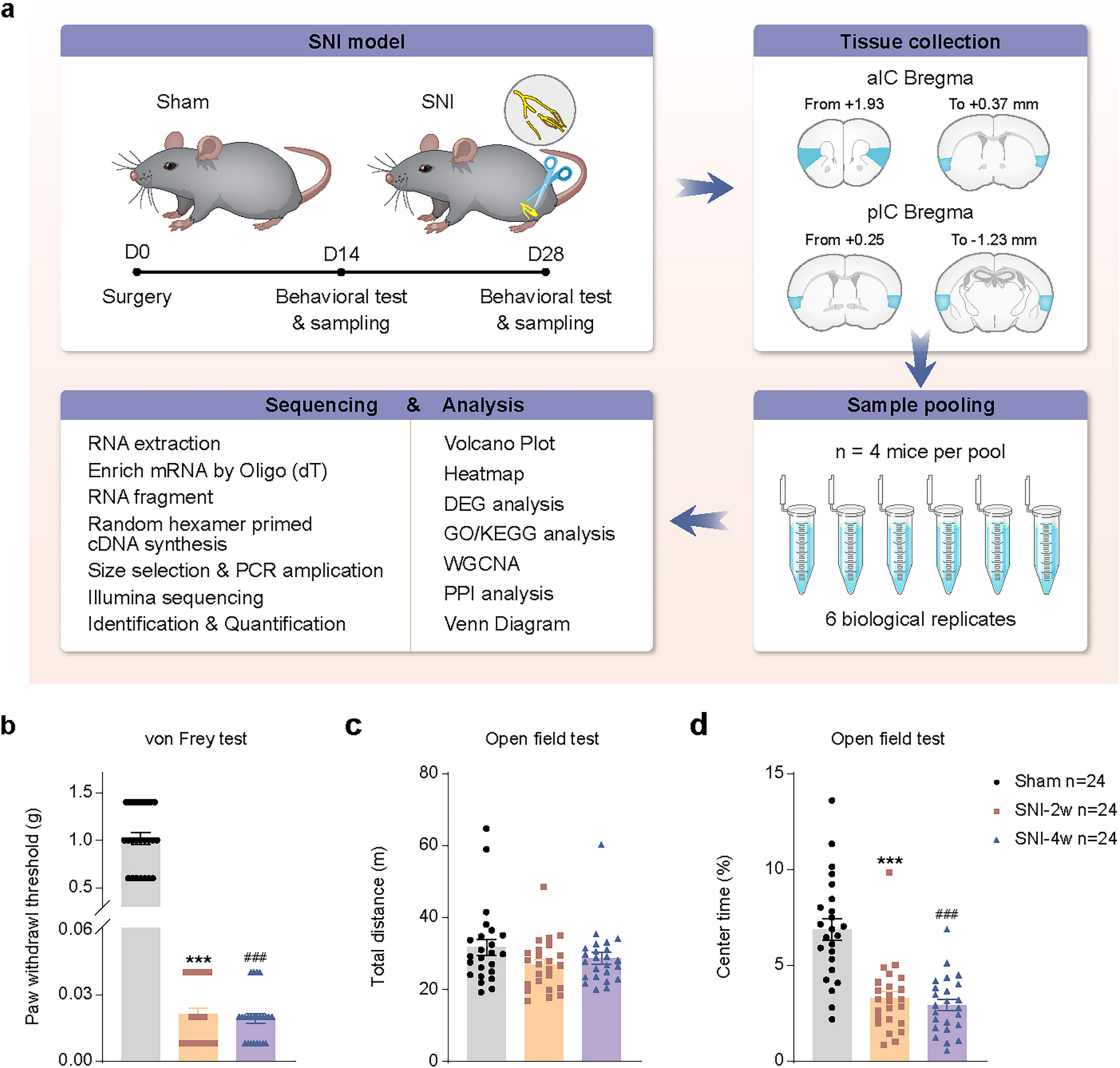
Systematic workflow and behavioral tests. **(a)** Transcriptome analysis workflow in the insular cortex of neuropathic pain mice. **(b–d)** SNI induced mechanical hyperalgesia and anxiety-like behaviors. SNI mice showed decreased paw withdrawal thresholds **(b)**, unchanged open field distance **(c)**, but reduced center time **(d)** compared to shams. One-way ANOVA. ***P < 0.001, SNI-2w vs. sham; ^###^*P* < 0.001, SNI-4w vs. sham.

Total RNA was extracted separately from the aIC (bregma +1.39 to +0.37 mm) and pIC (bregma +0.25 to –1.23 mm). For each region and experimental group, six independent biological replicate RNA pools were prepared. Each pool consisted of bilateral insular tissue combined from four animals (Figure 1a), yielding approximately 40 mg of aIC or pIC tissue per biological replicate (each animal contributed about 10 mg of aIC and pIC tissue), which was sufficient for subsequent RNA sequencing. All dissected tissue samples were stored at –80°C until further processing.

### 2.4 RNA sequencing

Total RNA was isolated from tissue samples using TRIzol reagent (Thermo Fisher Scientific, CA, United States). RNA quality was assessed with an Agilent 5300 Bioanalyzer, and concentration was determined using a NanoDrop 2000 spectrophotometer. Only RNA samples meeting the following criteria were used for library preparation: minimum total quantity of 1 μg, concentration ≥ 30 ng/μL, RNA Integrity Number > 6.5, and an OD260/280 ratio between 1.8 and 2.2. mRNA was enriched from qualified total RNA using Dynabeads Oligo(dT) (Thermo Fisher Scientific) and subsequently fragmented via incubation with divalent cations (Magnesium RNA Fragmentation Module, New England Biolabs). The fragmented mRNA was reverse-transcribed into first-strand cDNA using SuperScript™ II Reverse Transcriptase (Invitrogen). Second-strand cDNA synthesis was then performed in the presence of dUTP to generate U-labeled double-stranded DNA, employing a mixture of *E. coli* DNA polymerase I, RNase H, and dUTP Solution (Thermo Fisher Scientific and New England Biolabs). After end repair and A-tailing, the cDNA fragments were ligated to uniquely indexed adapters. Adapter-ligated products were size-selected using AMPure XP beads. To ensure strand specificity, libraries were treated with the heat-labile uracil-DNA glycosylase (UDG, New England Biolabs) to selectively degrade the U-labeled strand prior to polymerase chain reaction (PCR) amplification. PCR was conducted under the following thermal cycling conditions: initial denaturation at 95°C for 3 min; 8 cycles of denaturation at 98°C for 15 s, annealing at 60°C for 15 s, and extension at 72°C for 30 s; followed by a final extension at 72°C for 5 min. The final cDNA libraries, with an average insert size of 300 ± 50 bp, were subjected to paired-end sequencing (2 × 150 bp) on an Illumina NovaSeq X Plus platform.

### 2.5 Transcriptomic analysis

RNA sequencing generated approximately 870.41 million paired end reads (2 × 150 bp). Raw reads were initially processed using fastp (v0.22.0) to trim adapter sequences and filter low-quality bases, with subsequent quality evaluation performed by FastQC (v0.11.9).

Following quality control, a total of 259.77 Gb of high-quality clean reads were obtained. These reads were aligned to the mouse reference genome (GRCm39) using HISAT2 (v2.2.1). Gene-level quantification was performed with featureCounts (v2.0.1). Raw read counts were normalized and transformed to log2 counts per million [log_2_(CPM + 1)] using the cpm function in the edgeR package (v4.4.0) to stabilize variance and handle zero counts for subsequent expression analyses.

Principal component analysis (PCA) was conducted on the variance-stabilized expression matrix using the PCA function from the FactoMineR package (v2.11) in R. The first two principal components were visualized with the fviz_pca_ind function from the factoextra package (v1.0.7), with sample points colored by experimental group.

Differential gene expression (DEGs) analysis was performed using DESeq2 (v1.48.1). Genes were considered differentially expressed if they met the thresholds of | log_2_ fold change| (| (log_2_FC)|) > 0.58 and adjusted *p*-value (padj) < 0.05. The FC threshold was determined based on a review of recent transcriptomic studies on pain-related cortical changes in rodents, in which reported FC values typically ranged from 1 to 2 (Su et al., 2021; Dai et al., 2022; Zhang Y. et al., 2022; Qiu et al., 2023; Kang et al., 2024; Erdei et al., 2025; Xie et al., 2025). Therefore, the use of a moderate | log2FC| > 0.58 threshold, combined with rigorous padj < 0.05 correction, was intended to achieve a reasonable balance between maximizing the capture of true biological signals and controlling the risk of false positives.

We evaluated cell type-specific enrichment of our DEGs using the dataset from Zhang et al. (2014), which defines selectively enriched transcripts in mouse cortical neurons, glia and vascular cells. Enrichment scores were calculated from Fragments Per Kilobase of transcript per Million mapped reads (FPKM) values for each cell type (astrocytes, endothelial cells, neurons, microglia and oligodendrocytes) as: FPKM in target cell type divided by FPKM in all other cell types. A score > 1.5 indicated enrichment in that cell type.

Venn diagrams illustrating overlaps of DEGs across comparisons were generated with the ggVennDiagram package (v1.5.3). Functional enrichment analysis of DEGs for Gene Ontology (GO) terms and Kyoto Encyclopedia of Genes and Genomes (KEGG) pathways was conducted using the clusterProfiler R package (v4.16.0).

To construct a gene co-expression network, we utilized the Weighted Gene Co-expression Network Analysis (WGCNA) package (v1.73) to compute pairwise similarity between gene expression profiles (Langfelder and Horvath, 2008). A scale-free network was established following the WGCNA framework. The adjacency matrix, derived from expression correlations, was transformed into a topological overlap matrix (TOM) to better capture network interconnectedness. Hierarchical clustering was then performed on the TOM-based dissimilarity measure, and distinct gene modules were identified by applying a dynamic tree-cutting algorithm. Key parameters for network construction were set as follows: the minimum module size was set to 50 genes (minModuleSize = 50); the option to reassign genes between modules based on silhouette width was disabled (pamRespectsDendro = FALSE); and the dendrogram cut height for merging similar modules was set to 0.3 (cutHeight = 0.3).

To investigate functional interactions among DEGs, protein–protein interaction (PPI) networks were constructed using the STRING database (v12.0) (Szklarczyk et al., 2019) for significant DEGs identified in each subregion and time point. The analysis was conducted with the minimum required interaction confidence score set to “high confidence” (0.7), and isolated nodes were excluded from the resulting network visualizations. Hub DEGs and their associated sub-networks were subsequently identified based on the Maximal Clique Centrality score calculated using the cytoHubba plugin. All network data were visualized and further analyzed using Cytoscape software (v3.7.0) (Dai et al., 2022).

## 3 Results

### 3.1 Behavioral evaluation of mice following SNI

Behavioral tests were performed 2 weeks after surgery in the sham and SNI-2w groups, and 4 weeks after surgery in the SNI-4w group, to confirm the establishment of neuropathic pain models. Compared with the sham group, SNI-treated rats showed a marked reduction in PWT in the contralateral hind paw at both 2 and 4 weeks post-surgery (Figure 1b). In the open field test, SNI mice spent significantly less time in the central area 2 weeks after surgery; however, their total traveling distance was not significantly altered (Figures 1c,d). Together, these behavioral data demonstrate nociceptive hypersensitivity and anxiety-like phenotypes in SNI-treated rats.

### 3.2 Overview of RNA-seq data and basic alignment metrics

Transcriptomic profiling of insular cortex subregions was conducted using RNA sequencing at 14 and 28 days post-SNI or 14 days after sham surgery (Figure 1a). After quality filtering, an average of 23.98 million clean reads per sample were retained from approximately 24 million raw reads. Alignment to the mouse reference genome yielded mapping rates ranging from 98.5 to 98.9% (Supplementary Table 1). The distribution of detected genes across all samples is shown in Supplementary Figures 1a,b. To assess sample quality and experimental reproducibility, we calculated pairwise Pearson correlation coefficients between samples using normalized expression values. The resulting correlation matrix is displayed as a heatmap in Supplementary Figure 1c, confirmed high reproducibility among biological replicates.

### 3.3 Altered transcriptional signatures in the insular cortex after SNI

**FIGURE 2:**
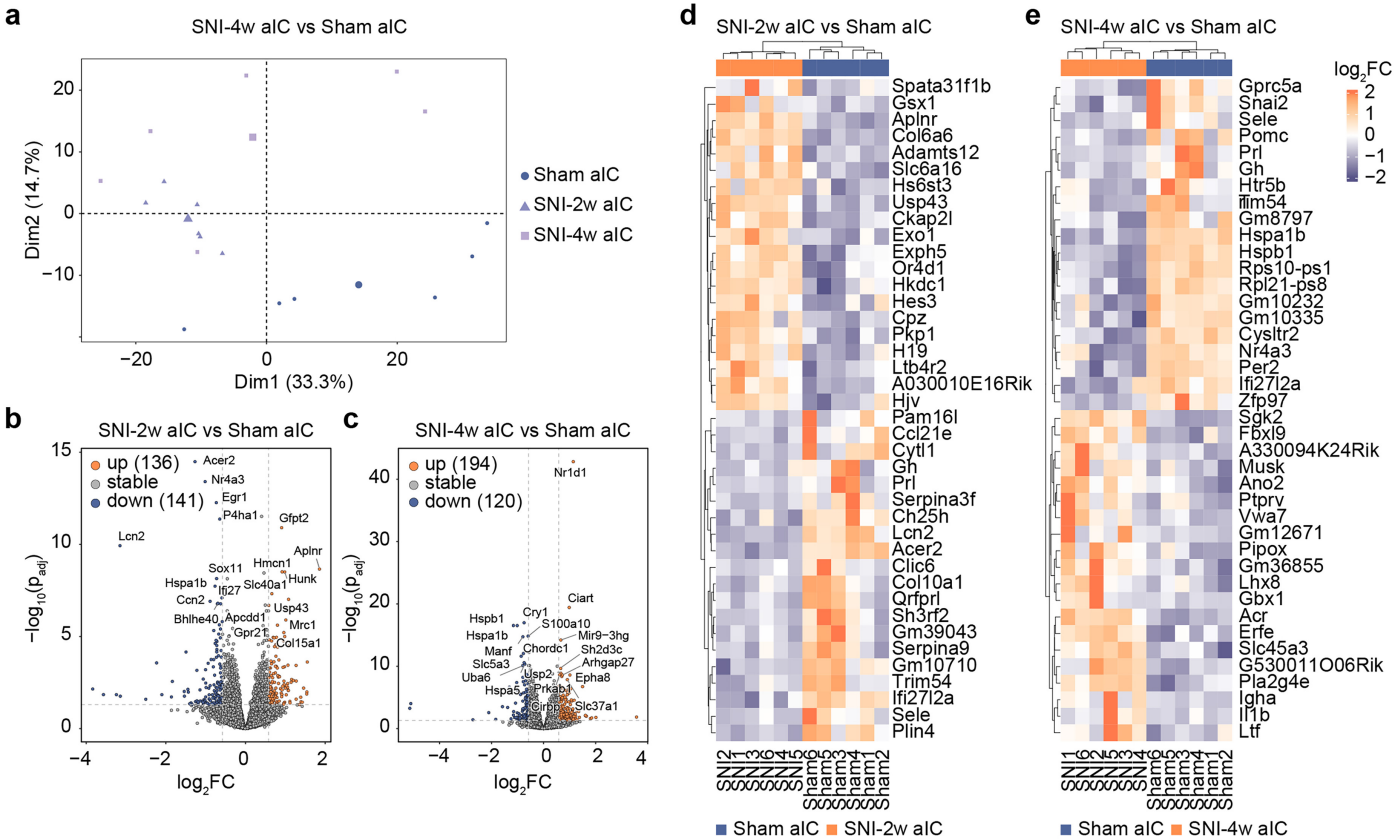
Overview of transcriptomic alterations in the aIC following SNI. **(a)** Principal component analysis illustrating distinct clustering of gene expression profiles across the three experimental groups. **(b,c)** Volcano plots depicting DEGs in the SNI-2w **(b)** and SNI-4w **(c)** cohorts relative to sham controls. Significantly up-regulated genes are highlighted in red, and down-regulated genes in blue. **(d,e)** Hierarchical clustering heatmaps of DEGs in the SNI-2w **(d)** and SNI-4w **(e)** groups versus sham. Red and blue shades denote up-regulation and down-regulation, respectively, with dendrograms representing gene clustering patterns. The color scale corresponds to Log_2_FC values.

**FIGURE 3:**
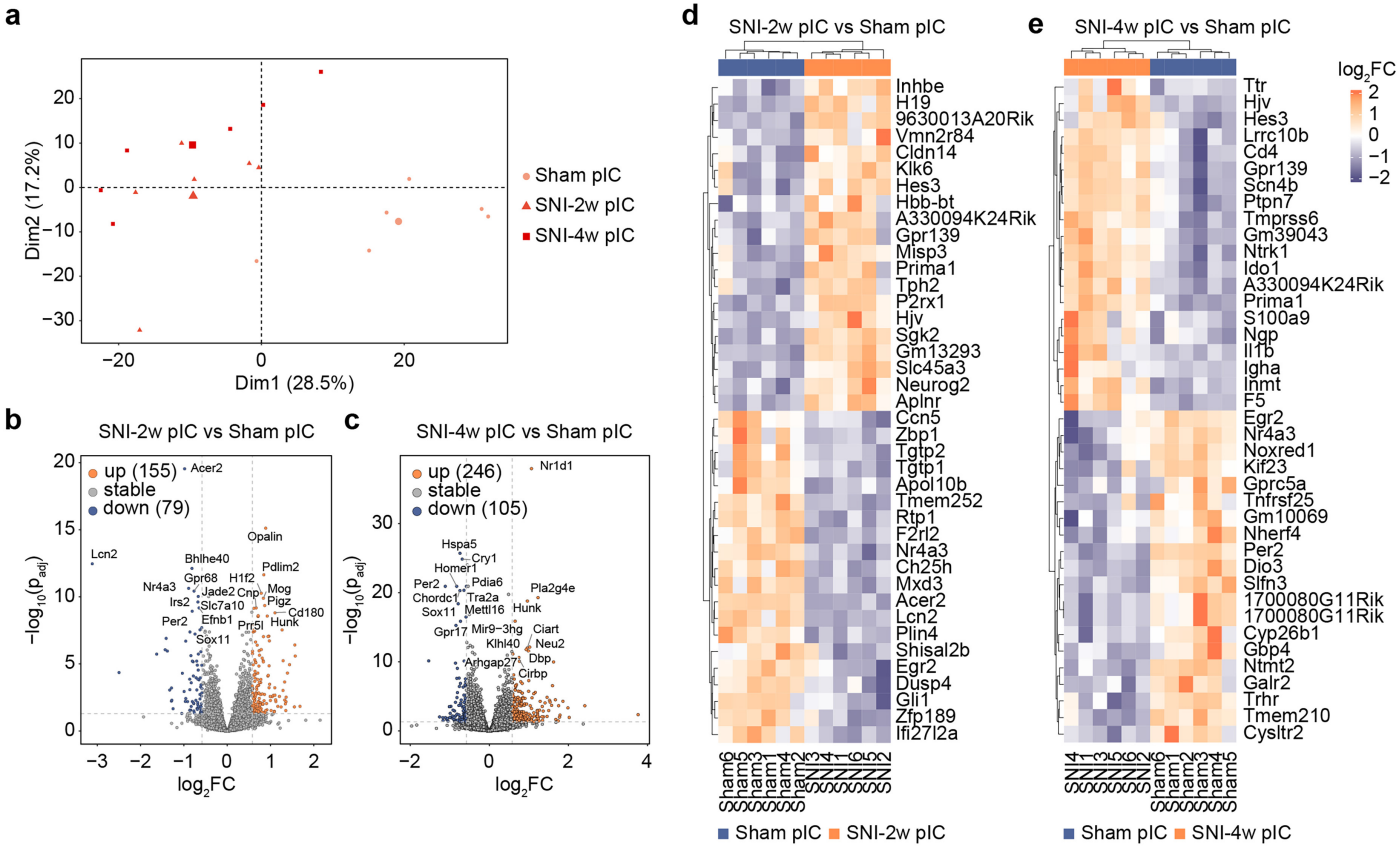
Overview of Transcriptomic Alterations in the pIC Following SNI. **(a)** Principal component analysis illustrating distinct clustering of gene expression profiles across the three experimental groups. **(b,c)** Volcano plots depicting DEGs in the SNI-2w **(b)** and SNI-4w **(c)** cohorts relative to sham controls. Significantly up-regulated genes are highlighted in red, and down-regulated genes in blue. **(d,e)** Hierarchical clustering heatmaps of DEGs in the SNI-2w **(d)** and SNI-4w **(e)** groups versus sham. Red and blue shades denote up-regulation and down-regulation, respectively, with dendrograms representing gene clustering patterns. The color scale corresponds to Log_2_FC values.

To characterize injury-induced transcriptional alterations across insular subregions, we performed differential expression analysis following SNI (Supplementary Data 1). At 2 weeks post-SNI, transcriptomic profiling identified approximately 38,513 and 38,605 expressed genes in the aIC and pIC, respectively. By 4 weeks, these numbers increased to 38,978 and 38,058 in the aIC and pIC, respectively. PCA revealed clear separation among the three experimental groups in both insular subregions, indicating distinct transcriptional states across conditions (Figures 2a, 3a). The number of DEGs increased in a time-dependent manner. In the aIC, 136 genes were upregulated and 141 downregulated at 2 weeks post-SNI relative to sham controls, whereas at 4 weeks these counts shifted to 194 upregulated and 120 downregulated genes (Figures 2b,c). Similarly, in the pIC we identified 155 upregulated and 79 downregulated DEGs at 2 weeks, increasing to 246 upregulated and 105 downregulated genes by 4 weeks (Figures 3b,c). Unsupervised hierarchical clustering of the top 40 DEGs (ranked by adjusted *P* value) confirmed clear separation between SNI and sham groups in both subregions and at both time points (Figures 2d,e, 3d,e). Across all comparisons, protein-coding transcripts accounted for most expression changes (85.5–91.1%), followed by long non-coding RNAs (4.2–11.8%) (Supplementary Figure 1d).

Using a cell-type-specific transcript database (Zhang et al., 2014), we quantified DEGs enriched in five cell types in the aIC and pIC at 2 and 4 weeks after SNI (Supplementary Figure S2 and Supplementary Data 2). Neurons exhibited substantial transcriptional changes in most comparisons, except in the pIC at 2 weeks after SNI, where only 9 DEGs were detected. Across other cell types, oligodendrocytes in the pIC showed the most striking upregulation (45 DEGs at 2 weeks, 33 at 4 weeks), whereas endothelial cells and microglia in the aIC also exhibited prominent changes, particularly at the early time point. Collectively, these region- and cell-type-specific dynamics may provide cellular insights into insular cortex mechanisms in chronic pain.

### 3.4 Dysregulation of pain-related signaling molecules, transcription factors and epigenetic regulators in the insular cortex

**FIGURE 4:**
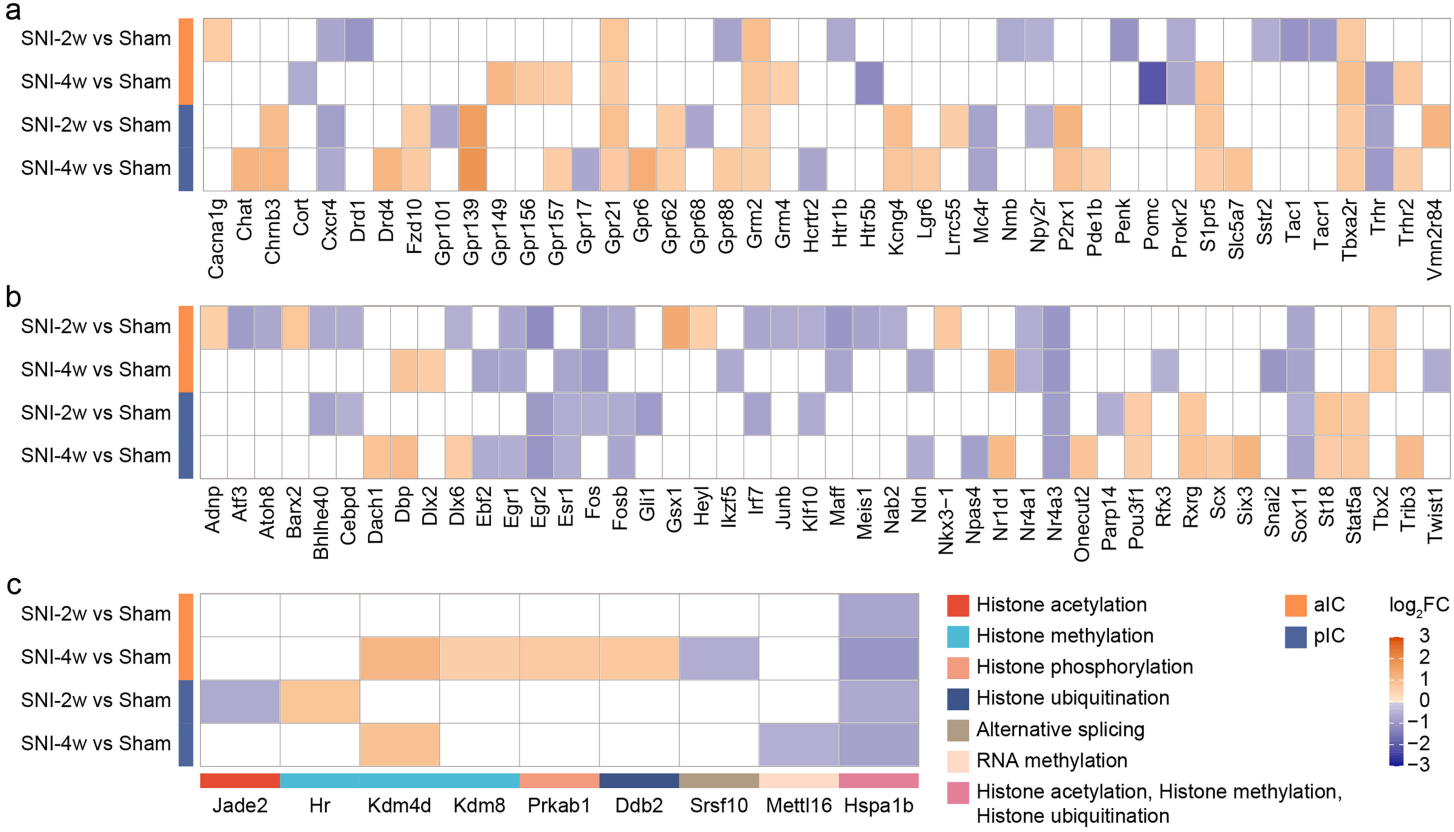
Heatmaps of the representative DEGs in the IC after nerve injury. **(a–c)** DEGs of synapse-related molecules, G protein-coupled receptors, ion channels, neuropeptides **(a)**, transcription factors **(b)**, and epigenetic regulators **(c)** in the IC after nerve injury. Colors in the heatmaps indicate the Log_2_FC among the different datasets. The up- and down-regulated genes are colored in red and blue, respectively. Under **(c)**, the bottom-row annotation designates the functional categories of epigenetic modifiers.

Synapse-related molecules, G protein-coupled receptors (GPCRs), ion channels, and neuropeptides play fundamental roles in nociceptive transmission and modulation (Campbell and Smrcka, 2018; Neugebauer et al., 2020; Bouali-Benazzouz et al., 2021). Drawing on existing functional annotations (Hashikawa et al., 2020; Wang J. et al., 2023), we compiled a reference gene set spanning these categories (Supplementary Table 2) and intersected it with our DEGs. This analysis revealed marked enrichment of DEGs encoding GPCRs, as well as representation of neuropeptides, ion channels, and a limited number of synapse-associated molecules (Figure 4a). Among neuropeptides showing altered expression, we identified *Tac1*—a known modulator of pain at spinal and supraspinal levels (Barik et al., 2018; Huang et al., 2019)—and *Prok2*, previously implicated in NP and affective dysfunction via actions in the nucleus accumbens shell (Wang et al., 2024). We also observed altered expression of Penk and Pomc, both well-characterized contributors to pain processing (Raffin-Sanson et al., 2003; Sapio et al., 2020; Ray et al., 2023). Within the GPCR class, several receptors with established roles in nociception exhibited differential expression. These included neuropeptide receptors *Mc4r* (Li et al., 2017; Sharfman et al., 2022; Ai et al., 2025) and *Npy2r* (Yan et al., 2022), the tachykinin receptor *Tacr1* (Choi et al., 2020; Li et al., 2025), the dopamine receptor *Drd1* (Bao et al., 2021; Huang M. et al., 2022), the metabotropic glutamate receptor *Grm2* (Chen et al., 2020), the chemokine receptor *Cxcr4* (Luo et al., 2016), as well as additional GPCRs such as *Gpr17* (Li et al., 2026) and *Gpr139* (Kononoff et al., 2018). Among ion channels, we detected altered expression of *Cacna1g*, which encodes a voltage-gated calcium channel subunit previously implicated in thalamic pain regulation (Jin et al., 2022). Furthermore, we observed expression changes in genes involved in acetylcholine metabolism—including *Chat* and *Slc5a7* in the pIC—consistent with previous reports highlighting the role of cholinergic transmission in pIC-mediated pain modulation (Ferrier et al., 2015).

Accumulating evidence highlights the involvement of transcription factors (TFs) in the pathogenesis of chronic pain (Masuda et al., 2014; Zhang et al., 2019; Pol, 2021). Here, we reveal an expanded repertoire of TFs exhibiting altered expression in the IC under NP conditions (Figure 4b), several of which have previously been implicated in CNS pain modulation. These include *Irf7* (a member of the interferon regulatory factor family), the stress-induced nuclear receptor *Nr4a1*, and the development-associated factor *Sox11*. Each has been implicated in spinal pain regulation via neuroinflammatory mechanisms or modulation of GABAergic transmission (Jiang et al., 2024; Le et al., 2024; Deng et al., 2025). In addition, the nuclear receptor superfamily member *Esr-1* contributes to pain-related aversion by regulating excitatory synaptic transmission in the anterior cingulate cortex (ACC) (Zang et al., 2020). Notably, all of these genes were downregulated in the NP model. Another prominent category of down-regulated TFs comprises immediate-early genes (IEGs), all of which showed reduced expression under NP conditions. These include *Egr1*, *Egr2*, *Fos*, *Fosb*, *Junb*, and *Npas-4*, all of which participate in chronic pain formation within the cerebral cortex or spinal cord (Niikura et al., 2011; Zhuo, 2011; Wang et al., 2019; Wu et al., 2019). In contrast, we observed elevated expression of *Adnp*, a protein with established roles in brain development and function. While no prior studies have directly linked it to pain mechanisms, existing evidence indicates that it regulates synaptic plasticity in ACC neurons via Wnt signaling and mitigates anesthesia-induced social and cognitive impairments (Liang et al., 2024).

Broad transcriptional changes are often associated with extensive chromatin remodeling and epigenetic regulation, which play critical roles in pain development (Doehring et al., 2011). Among 720 known epigenetic regulators (Medvedeva et al., 2015), we identified dysregulation of a specific subset of histone-modifying genes across insular subregions in SNI mice relative to sham controls (Figure 4c). These included genes involved in histone acetylation (*Jade2*), histone methylation (*Hr, Kdm4d, Kdm8*), histone phosphorylation (*Prkab1*), histone ubiquitination (*Ddb2*), and multiple histone modifications (*Hspa1b*). Additionally, we observed expression changes in *Srsf10*, related to alternative splicing, and *Mettl16*, involved in RNA methylation. Of note, *Hspa1b*—encoding a member of the heat shock protein A family—has been implicated in nociceptive modulation. Emerging evidence suggests that Hspa1b may contribute to NP through mechanisms involving endoplasmic reticulum (ER) stress in the SDH (Wang Q. et al., 2023).

### 3.5 Functional enrichment analysis of the differentially expressed genes after SNI

**FIGURE 5:**
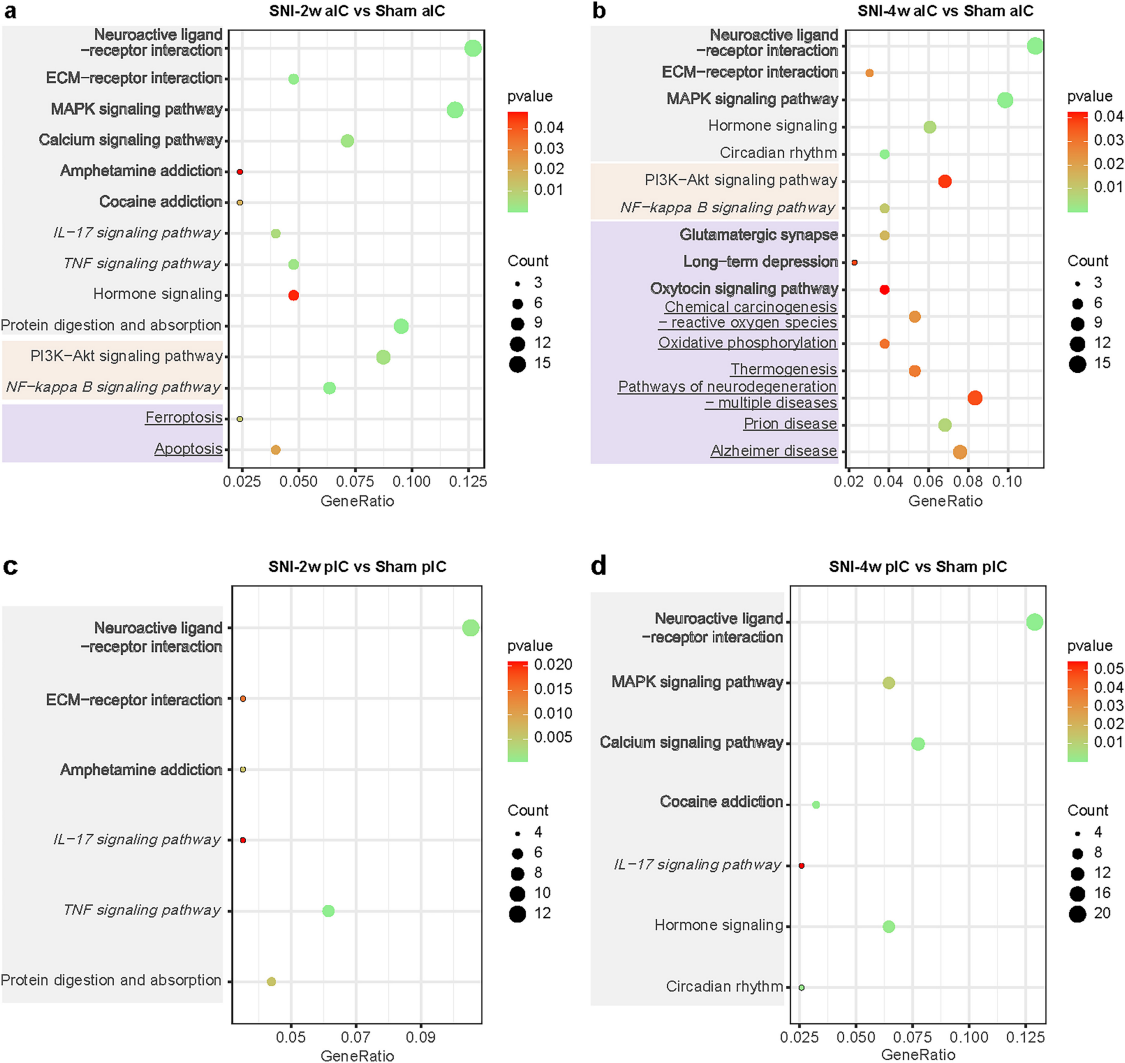
KEGG enrichment of DEGs in insular cortex after SNI. **(a–d)** Bar plots for aIC (SNI-2w vs. Sham), aIC (SNI-4w vs. Sham), pIC (SNI-2w vs. Sham), and pIC (SNI-4w vs. Sham), respectively. Bold, italic, underlined and plain text denote pathways related to synaptic transmission/plasticity, neuroinflammation, oxidative stress or other processes, respectively. Gray, pink and purple boxes indicate pathways shared between aIC and pIC, shared between the two aIC time points, and unique to individual aIC time points, respectively. Bubble size represents the number of genes per pathway, and color indicates the *p*-value.

To delineate the functional consequences of the DEGs, KEGG pathway enrichment analysis was performed using thresholds of p < 0.05 and a minimum gene count of 2 per pathway (Supplementary Data 3). Under these criteria, 47, 29, 23 and 17 pathways were significantly enriched in the aIC SNI-2w versus Sham, aIC SNI-4w versus Sham, pIC SNI-2w versus Sham, and pIC SNI-4w versus Sham comparisons, respectively. The principal results are summarized in Figure 5. Collectively, the enriched pathways spanned three broad functional domains: (1) neural signaling and synaptic plasticity, represented by neuroactive ligand–receptor interaction, mitogen-activated protein kinase (MAPK), Extracellular matrix (ECM)-receptor interaction, and calcium signaling; (2) immune–inflammatory responses, represented by tumor necrosis factor (TNF) and interleukin-17 (IL-17) signaling pathways; and (3) a core axis of oxidative stress, mitochondrial dysfunction and programmed cell death (ferroptosis/apoptosis), which collectively forms the molecular basis of neurodegeneration. Other enriched pathways included Phosphoinositide 3-kinase-protein kinase B (PI3K-Akt) signaling and circadian rhythm, among others.

From a subregional perspective of the IC, several pathways related to synaptic transmission/plasticity, neuroinflammation, and other processes (including circadian rhythm, hormone signaling, protein digestion and absorption) were conserved across both time points in both the aIC and pIC. Some pathways were unique to the aIC. PI3K-Akt signaling and NF-κB signaling were enriched at both time points, whereas programmed cell death-related pathways were enriched only at the early time point, and certain synaptic transmission/plasticity-related pathways (glutamatergic synapse, long-term depression, oxytocin signaling pathway) appeared exclusively at the late time point. Notably, pathways associated with oxidative stress, mitochondrial dysfunction, and neurodegeneration were enriched only at the late time point.

**FIGURE 6:**
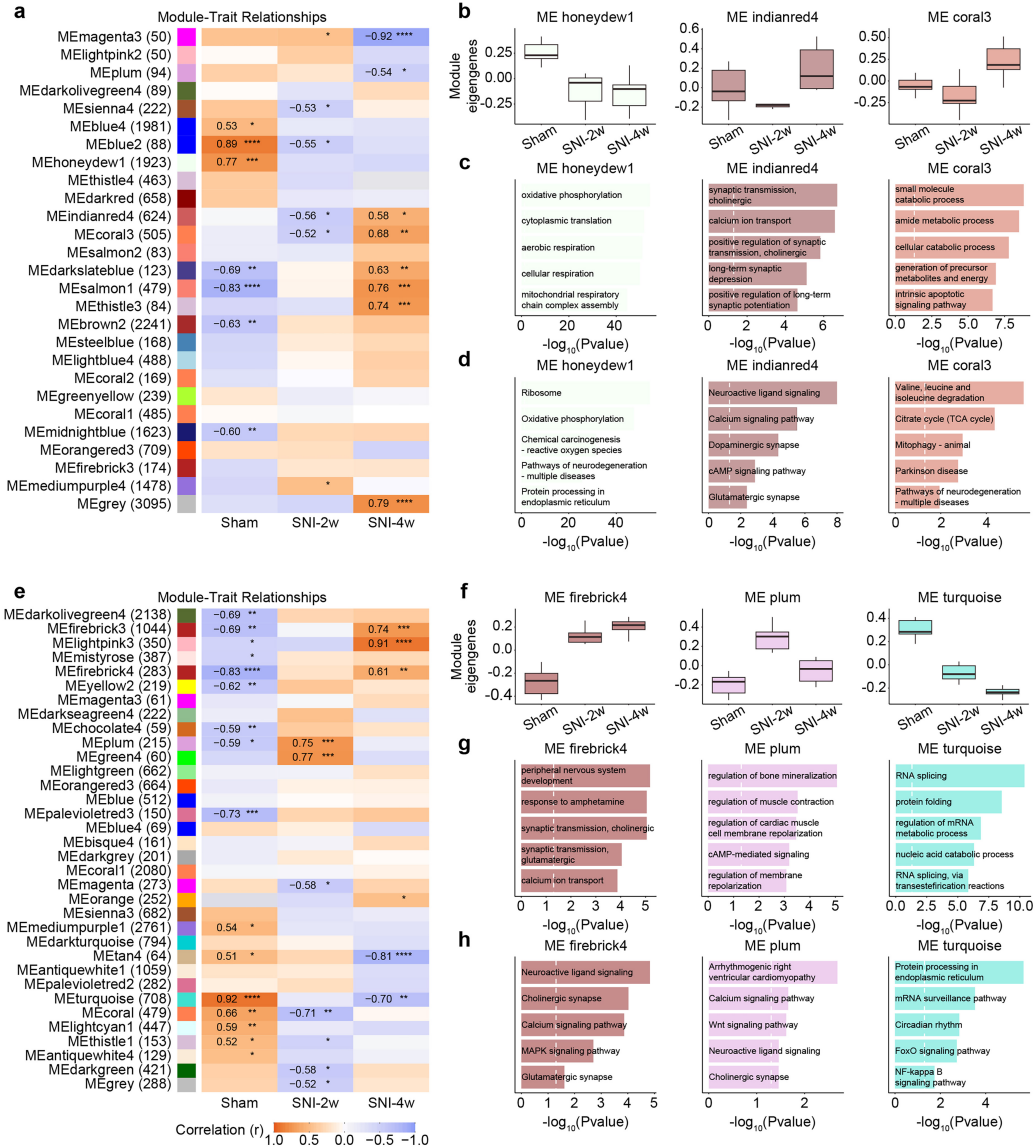
Weighted gene co expression network analysis. **(a)** Heatmap showing the correlation between co expression modules identified in the aIC and experimental groups (sham, SNI-2w, SNI-4w). Each cell indicates the correlation coefficient (r) and corresponding *P*-value for a module-phenotype pair. Positive values (*r*> 0) reflect module up-regulation, negative values (*r*> 0) indicate down-regulation, with color intensity scaled to the magnitude of correlation. Statistical thresholds are marked as follows: *P < 0.05, **P < 0.01, ***P < 0.001, ****P < 0.0001. **(b)** Distribution of module eigengene expression (mean log_2_CPM) across groups, presented as boxplots. **(c,d)** Functional enrichment results for three modules highlighted in a. Representative GO biological process terms **(c)** and KEGG pathways **(d)** are displayed, organized by experimental group (sham, SNI-2w, SNI-4w from left to right). **(e-h)** Corresponding WGCNA results for the pIC, following the same layout and conventions as **(a-d)**.

To extend our characterization of DEGs, we performed WGCNA separately for the anterior and posterior insular cortices (Figure 6 and Supplementary Figure 3). Network construction employed a soft-thresholding power (β) of 3 to satisfy scale-free topology criteria (*R*^2^ > 0.8). Modules were defined using topological overlap matrices with dynamic tree-cutting (minimum module size = 50). Modules exhibiting high similarity (cut-height = 0.3) were merged, resulting in 27 distinct co-expression modules in the aIC (sizes ranging from 50 genes in “magenta3” to 3,095 genes in “gray”; Figure 6a) and 34 modules in the pIC (sizes from 60 genes in “green4” to 2,761 genes in “mediumpurple1”; Figure 6e). To assess the functional relevance of aIC modules, we correlated module eigengenes with experimental conditions. Several modules displayed significant associations with neuropathic pain states (Figures 6a,b). Specifically, the honeydew1 module correlated positively with the sham group (*r* = 0.77, *P* < 0.001), whereas indianred 4 was negatively correlated with the SNI-2w group (*r* = –0.56, *P* < 0.05). The coral3 module demonstrated a strong positive correlation with the SNI-4w group (*r* = 0.68, *P* < 0.01). These three modules were consequently prioritized for downstream enrichment analysis. GO biological process and KEGG pathway analyses revealed distinct functional profiles. Honeydew1 was enriched for terms linked to “Oxidative phosphorylation,” “Protein processing in endoplasmic reticulum,” and “Pathways of neurodegeneration—multiple diseases.” The indianred4 module exhibited enrichment in synaptic-transmission and plasticity-related pathways such as “Neuroactive ligand–receptor interaction,” “Calcium signaling pathway,”, and “Glutamatergic synapse.” The Coral3 module was associated with cellular metabolic processes, including “Mitophagy,” “Parkinson disease”, and “Pathways of neurodegeneration—multiple diseases” (Figures 6c,d).

Likewise, in the pIC, three key co-expression modules showed significant correlations with experimental conditions (Figures 6e,f). Specifically, the firebrick4 module correlated negatively with the sham group (*r* = –0.83, *P* < 0.0001), the plum module showed a positive correlation with the SNI-2w group (*r* = 0.75, *P* < 0.001), and the turquoise module exhibited a negative correlation with the SNI-4w group (*r* = –0.70, *P* < 0.01). Functional enrichment analysis revealed distinct pathway associations. The firebrick4 module was enriched in synaptic transmission-related processes, including “Neuroactive ligand–receptor interaction,” “Calcium signaling pathway,” and “Glutamatergic synapse.” The plum module was also associated with synaptic signaling pathways, namely “Neuroactive ligand–receptor interaction,” “Calcium signaling pathway,” and “Cholinergic synapse.” In contrast, the turquoise module was linked to protein-folding and processing, particularly involving “Protein processing in endoplasmic reticulum” (Figures 6g,h).

Together, these WGCNA findings corroborate our earlier KEGG pathway enrichment results, reinforcing the critical role of synaptic transmission and plasticity in NP pathophysiology. Moreover, the enrichment observed in pathways related to mitochondrial dysfunction and ER stress suggests that NP may share overlapping pathological mechanisms with several neurodegenerative conditions.

### 3.6 Cross-dataset validation between the insular and cingulate cortices

To externally validate the key transcriptomic signatures identified in the IC, we performed a parallel RNA-seq analysis on the cingulate cortex from the NP mouse cohort (NCBI Sequence Read Archive under the accession number PRJNA1417940). Here, we focused on two subregions of the cingulate cortex: the ACC and the midcingulate cortex (MCC). The ACC has long been established to be involved in both the sensory and affective dimensions of pain, whereas the MCC was initially thought to be primarily involved only in the sensory dimension (Tan et al., 2017; Kragel et al., 2018; Hu et al., 2019); however, emerging evidence suggests that the MCC also participates in the affective dimension of pain (Kisler et al., 2016; Hong et al., 2023; Li X. et al., 2023).

The ACC and MCC were profiled at 2 and 4 weeks after SNI using identical bioinformatics pipelines as for the IC (Supplementary Data 4). At 2 weeks post-SNI, 46 DEGs were commonly altered between the aIC and ACC; by 4 weeks, this number increased to 120 DEGs. Between the pIC and MCC, 25 overlapping DEGs were identified at 2 weeks and 64 at 4 weeks. These shared DEGs accounted for 16.6, 38.2, 10.7, and 18.2% of the total DEGs in the SNI 2w aIC, SNI 4w aIC, SNI 2w pIC, and SNI 4w pIC groups, respectively, and also contributed substantially to the DEG pools of the ACC and MCC. Thus, transcriptional reprogramming converged between the insular and cingulate cortices under NP conditions, with the overlap increasing over time. To determine whether the overlapping genes are enriched in functionally relevant pathways, we performed KEGG enrichment analysis on the four sets of overlapping DEGs (aIC/ACC at 2w and 4w; pIC/MCC at 2w and 4w). As summarized in Supplementary Data 4, the overlapping DEGs between aIC and ACC at 4 weeks post-SNI remained significantly enriched in pathways related to mitochondrial function and neurodegenerative diseases. Moreover, pathways associated with MAPK signaling, neuroactive ligand–receptor interaction, protein digestion and absorption, and circadian rhythm were consistently enriched across all four overlapping datasets. Together, these cross-dataset comparisons provide strong external validation for the key transcriptomic signatures we observed in the IC, reinforcing the central involvement of neuroinflammation, mitochondrial dysfunction, ER stress and synaptic plasticity in the cortical pathophysiology of NP.

### 3.7 Protein-protein interaction analysis after SNI

**FIGURE 7:**
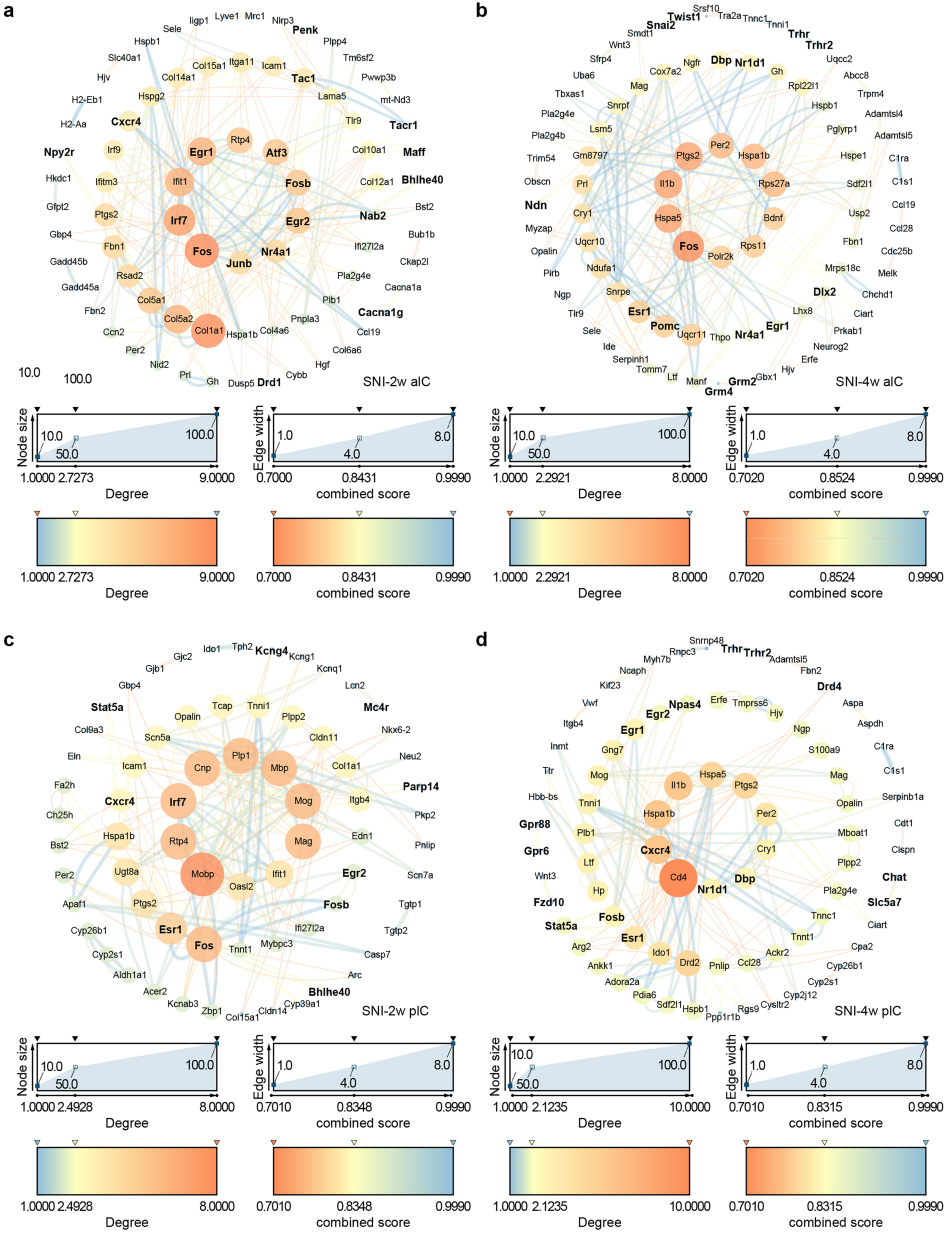
PPI networks of DEGs in SNI models relative to sham controls. **(a,b)** STRING-based PPI networks of DEGs identified in the aIC at 2 weeks **(a)** and 4 weeks **(b)** post-SNI. **(c,d)** Corresponding networks for the pIC at 2 weeks **(c)** and 4 weeks **(d)** after SNI. In each network, node color and size reflect connectivity degree (number of interactions), whereas edge color and thickness denote the composite interaction confidence score derived from the STRING database. The 10 most highly connected nodes in each network are highlighted as hub genes; remaining nodes represent other DEGs included in the predicted interactome. Genes annotated as synapse-associated molecules, G-protein-coupled receptors, ion channels, neuropeptides, or transcription factors are marked with bold labels in the corresponding panels.

To explore molecular interactions and pinpoint potential key regulators among DEGs in the aIC and pIC following SNI, we performed PPI network analysis using the STRING database. In the aIC, the PPI network of DEGs comprised 77 nodes with 210 edges in the SNI-2w group and 89 nodes with 204 edges in the SNI-4w group. The top ten hub genes in the SNI-2w network were predominantly interferon-response genes (e.g., *Ifit1*, *Irf7*, *Rtp4*) together with IEGs such as *Egr1*, *Fosb*, *Egr2*, *Junb*, *Fos* and *Nr4a1*. In contrast, the top hub genes in the SNI-4w group were functionally more diverse, including circadian regulators (*Per2*), ER stress-related molecules (*Hspa1b*, *Hspa5*), inflammatory mediators (*Il1b*, *Ptgs2*), the IEG *Fos*, and the neurotrophic factor *Bdnf* (Figures 7a,b). Similarly, in the pIC, network analysis identified 69 nodes with 172 edges in the SNI-2w group and 81 nodes with 172 edges in the SNI-4w group. The ten most connected DEGs in the 2-week pIC network were largely core myelin components (*Plp1*, *Mbp*, *Mog*, *Mag*, *Mobp*, *Cnp*) together with interferon-response genes (*Ifit1*, *Irf7*, *Rtp4*, *Oasl2*). By contrast, hub genes in the SNI-4w pIC network again showed broader functional associations, encompassing circadian rhythm genes (*Per2*, *Cry1*, *Dbp*, *Nr1d1*), ER stress markers (*Hspa1b*, *Hspa5*), and inflammatory mediators (*Il1b*, *Ptgs2*, *Cd4*, *Cxcr4*) (Figures 7c,d). Collectively, these results imply that neuroinflammatory responses and ER stress may represent central mechanisms driving the pathological progression of NP within the IC. These PPI findings align with our KEGG and WGCNA results, further implicating ER stress and neuroinflammation as central drivers of NP pathogenesis in the IC.

### 3.8 Shared transcriptional changes across insular subregions and time points

**FIGURE 8:**
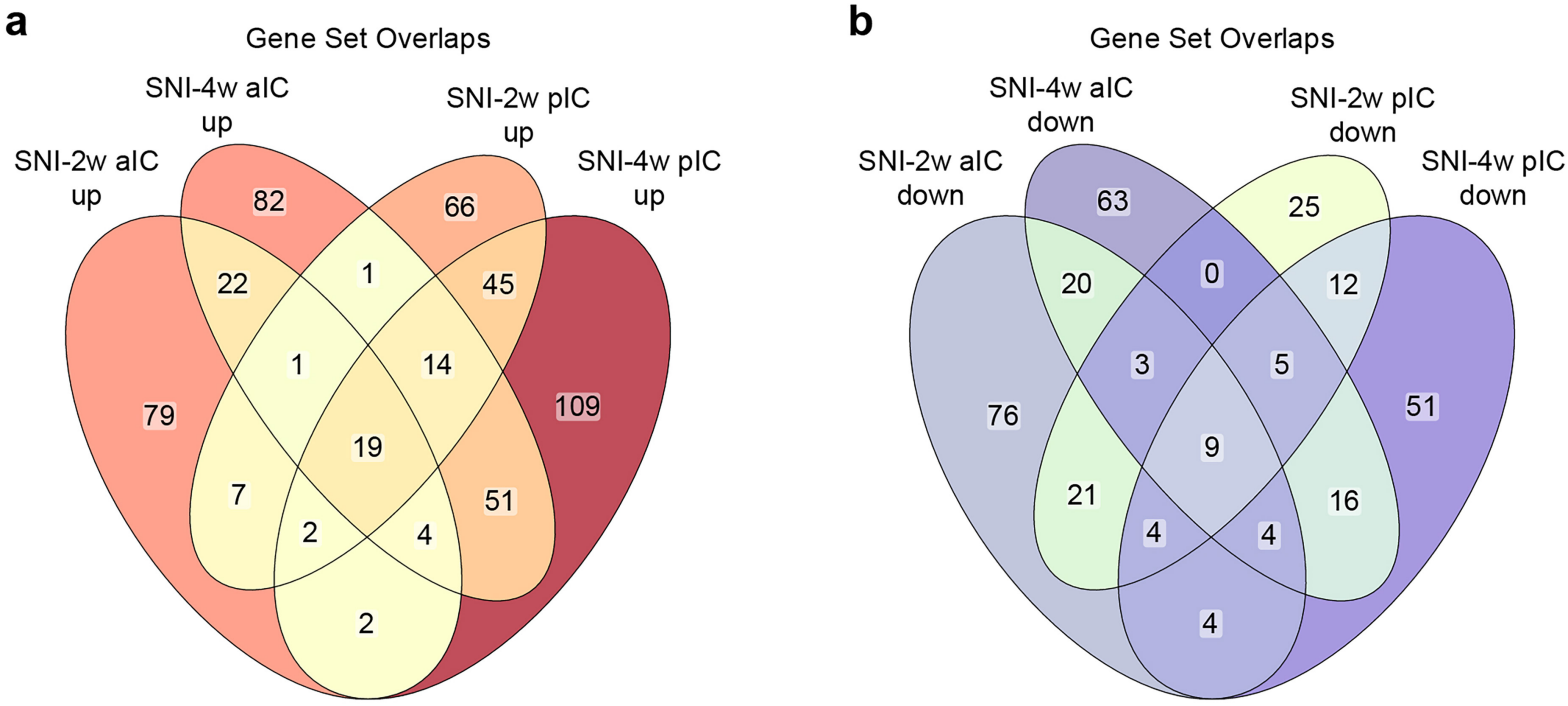
Integrated transcriptomic analysis. **(a,b)** Venn diagrams showing the partially overlapping sets of up-regulated **(a)** and down-regulated **(b)** DEGs identified in SNI-2w and SNI-4w groups relative to sham controls across insular subregions.

Integration of all transcriptomic datasets via Venn analysis identified 28 DEGs that were commonly dysregulated across both insular subregions and time points following SNI (Figure 8 and Table 1). Among these, 19 genes exhibited consistent up-regulation. To date, only *Grm2*, which encodes a group II metabotropic glutamate receptor, has previously been implicated in chronic pain at the CNS level. Up-regulation of *Grm2* in the SDH and ACC has been associated with analgesic effects in chronic pain models, potentially through inhibition of excitatory neurotransmission within nociceptive pathways (Cao et al., 2015; Notartomaso et al., 2017; Chen et al., 2020). The remaining nine overlapping DEGs were consistently downregulated. Several of these, including *Ptgs2*, *Per2*, *Sox 11*, and *Hspa1b*, have established roles in chronic pain within the CNS, with the latter two discussed in preceding sections. Notably, *Per2* and *Ptgs2* have been implicated in NP through neuroinflammatory mechanisms within the SDH (Chen et al., 2023; Yamakawa et al., 2024).

**TABLE 1:** Overlapping up-regulated and down-regulated DEGs in the insular subregions at 2 and 4 weeks post-SNI, compared to sham controls.

Gene symbol	Gene name
Downregulated genes	
Nr4a3	Nuclear receptor subfamily 4 group A member 3
Dusp4	Dual specificity phosphatase 4
Ptgs2	Prostaglandin-endoperoxide synthase 2
Glra3	Glycine receptor alpha 3
Per2	Period circadian regulator 2
Sox11	SRY-box transcription factor 11
Gbp4	Guanylate binding protein 4
Hspa1b	Heat shock protein 1B
2500002B13Rik	RIKEN cDNA 2500002B13 gene
Upregulated genes	
Sgk2	Serum/glucocorticoid regulated kinase 2
Gfpt2	Glutamine-fructose-6-phosphate transaminase 2
Npc1l1	NPC1 like intracellular cholesterol transporter 1
Usp43	Ubiquitin specific peptidase 43
Cd180	CD180 molecule
Grm2	Glutamate metabotropic receptor 2
Neurog2	Neurogenin 2
Map3k21	Mitogen-activated protein kinase kinase kinase 21
Tbxa2r	Thromboxane A2 receptor
Mag	Myelin-associated glycoprotein
Hjv	Hemojuvelin BMP co-receptor
Pigz	Phosphatidylinositol glycan anchor biosynthesis class Z
Tmem125	Transmembrane protein 125
Pkdrej	Polycystin family receptor for egg jelly
Gpr21	G protein-coupled receptor 21
Hunk	Hormonally up-regulated Neu-associated kinase
Atp10b	ATPase phospholipid transporting 10B
B3gnt9	UDP-GlcNAc:betaGal beta-1,3-N-acetylglucosaminyltransferase 9
Gm12992	Predicted gene 12992

## 4 Discussion

Although the cellular and circuit-level mechanisms by which the IC mediates NP have been progressively elucidated, its underlying molecular architecture remains poorly defined. Here, we address this gap by systematically profiling temporal transcriptomic dynamics of two IC subregions following prolonged peripheral nerve injury. We observed that the biological pathways enriched by DEGs were broadly conserved between the two major insular subdivisions. These data not only corroborate previously established roles for synaptic transmission and plasticity in NP but also reveal a novel pathogenic dimension: at the cortical level, NP shares fundamental pathophysiological mechanisms—such as neuroinflammation, proteostatic disruption, and mitochondrial dysfunction—with neurodegenerative disorders.

### 4.1 Synaptic plasticity mechanisms in the insular cortex underlying neuropathic pain

Chronic pain is sustained in part by central sensitization—a form of synaptic plasticity characterized by heightened neuronal responsiveness within central nociceptive pathways following injury. At the cortical level, nociceptive inputs are relayed via the thalamus to the ACC and IC, where they enhance synaptic plasticity and neuronal excitability in this region (Basbaum et al., 2009). Long-term potentiation of glutamatergic transmission in these areas represents a well-established model for studying the synaptic mechanisms underlying chronic pain (Bliss et al., 2016; Zhuo, 2016). Our transcriptomic profiling revealed enrichment of DEGs in synapse-related functions and pathways in the IC of NP mice compared with sham controls. These findings provide mechanistic support for the involvement of insular synaptic dysregulation in NP and reinforce the potential of these signaling cascades as therapeutic targets.

Chronic nociceptive input has been shown to induce presynaptic glutamate release within the IC. In parallel, postsynaptic expression of AMPARs and NMDARs is upregulated, a process that depends on the phosphorylation of the NMDAR subunit GluN2B and the AMPAR subunit GluA1. The cyclic adenosine monophosphate (cAMP) signaling pathway plays a key role in mediating these phosphorylation events (Qiu et al., 2013; Qiu et al., 2014; Bai et al., 2019; Li et al., 2024). Accordingly, selective pharmacological inhibition of cAMP signaling, Ca^2+^-permeable AMPARs, or GluN2B-containing NMDARs within the IC attenuates both behavioral hypersensitivity and associated negative affect in preclinical models of chronic pain (Qiu et al., 2013; Qiu et al., 2014; Li et al., 2024), underscoring their therapeutic potential for NP. Our transcriptomic data align with these observations, revealing significant enrichment of IC-derived DEGs in pathways central to synaptic modulation—including glutamatergic synapse, MAPK signaling, calcium signaling, and cAMP signaling pathways. A point worth noting is that, in the context of NP, the MAPK signaling pathway within the ACC—functionally analogous to the cAMP pathway—promotes pain-related negative affect by facilitating cAMP response element-binding protein phosphorylation. Accordingly, selective pharmacological inhibition of the MAPK pathway in the ACC attenuates both behavioral hypersensitivity and associated negative affect in preclinical models of chronic pain (Cao et al., 2014; Galan-Arriero et al., 2014). Although the MAPK pathway in the IC has been implicated in taste processing (Berman et al., 1998), its role in pain processing remains to be elucidated.

### 4.2 Converging pathways of neuropathic pain and neurodegeneration in the insular cortex

Beyond the well-established mechanisms of synaptic plasticity central to current research on IC-mediated chronic pain, our functional analyses also reveal previously understudied regulatory pathways. Neuroinflammation, characterized by glial activation and the release of proinflammatory cytokines and chemokines, contributes to central sensitization (Ji et al., 2018). At the cortical level, neuroinflammatory mechanisms have been well documented to contribute to the pathogenesis of chronic pain within the ACC. Sustained noxious stimuli activate cingulate astrocytes (Wei et al., 2024), resulting in elevated levels of inflammatory cytokines, including TNF-α. TNF-α potentiates cingulate synaptic transmission through presynaptic mechanisms and GABAergic disinhibition, collectively shifting the excitatory–inhibitory balance toward excitation (Jia et al., 2007; Duan et al., 2022). Moreover, increased TNF-α in the ACC activates the NF-κB pathway, leading to upregulation of acid-sensing ion channel 1a and consequent hyperexcitability of ACC glutamatergic neurons (Jiang et al., 2025). Accordingly, selective inhibition of TNF-α or NF-κB signaling attenuates behavioral hypersensitivity and negative affective states associated with chronic pain (Yao et al., 2019; Duan et al., 2022; Dai et al., 2025; Jiang et al., 2025). In contrast, neuroinflammatory mechanisms within the IC remain underexplored in chronic pain, with only limited evidence indicating glial activation and release of pro-inflammatory mediators such as TNF-α under NP conditions (Choi et al., 2022; Kim et al., 2023; Xu et al., 2023) . Consistent with these observations, the present study demonstrates that DEGs identified in the IC are significantly enriched in TNF and NF-κB signaling pathways, further highlighting the pivotal role of cortical neuroinflammation in NP pathophysiology.

The mitotoxicity hypothesis, initially derived from studies on chemotherapy-induced NP, has since been confirmed in peripheral nerve injury models. Such injuries trigger mitochondrial dysfunction in primary sensory neurons by impairing core processes—such as energy production, trafficking, fission/fusion dynamics, and mitophagy—and by increasing nitroxidative stress. These disruptions collectively contribute to neuronal sensitization (Bennett et al., 2014; Doyle and Salvemini, 2021). Mitophagy, the selective degradation of impaired mitochondria via autophagy, is a fundamental quality control mechanism. Dysregulation of this process in the peripheral nervous system results in the buildup of defective mitochondria, elevated reactive oxygen species, microglial activation, and demyelination, all of which facilitate NP progression (Dai et al., 2020; Huang Z. et al., 2022). Within the SDH, compromised mitophagy in microglia promotes NLR family pyrin domain containing 3 (NLRP3) inflammasome activation through mitochondrial ROS, thereby driving central pain plasticity (Shao et al., 2021). Reinforcing mitophagy has proven effective in alleviating pain in experimental models (Doyle and Salvemini, 2021; Shao et al., 2021). Recent evidence identifies impairment of the PINK1/PARKIN-mediated mitophagy pathway in the ACC of NP-affected rats. Notably, treadmill exercise restored mitochondrial function by reactivating this pathway, an effect that correlated with reduced pain-like behaviors (Li C. et al., 2023). These observations, together with the evidence of mitochondrial dysfunction within the IC presented in this study, highlight mitochondrial dysfunction at the cortical level as a significant but underinvestigated element in NP pathophysiology that warrants further exploration.

ER stress and the subsequent activation of the unfolded protein response have been implicated in the pathogenesis of chronic pain. ER stress disrupts multiple molecular cascades, including pro-inflammatory signaling, aberrant calcium handling, synaptic disinhibition, mitochondrial dysfunction, and ROS generation, as well as apoptosis. These disturbances collectively contribute to the hyper-excitability of nociceptive neurons through both inflammatory and non-inflammatory mechanisms. The synergistic interplay among these perturbed pathways may drive the exponential progression of pathological processes and promote the development of hyperalgesia. Markers of ER stress have been observed in both peripheral and central (spinal) neurons across various pain models. Notably, pharmacological inhibition of ER stress has been shown to ameliorate pain outcomes. Conversely, pharmacologically induced ER stress is sufficient to elicit hyperalgesia in naive animals (Yousuf et al., 2020; Kim et al., 2024; Liu et al., 2024). In the present study, we report for the first time alterations in ER stress-related genes within the IC, providing a mechanistic basis for the involvement of cortical ER stress in the NP pathophysiology.

Although traditionally viewed as distinct entities, chronic pain and neurodegenerative disorders are increasingly recognized for their significant clinical and pathophysiological overlap. On one hand, chronic pain is associated with cognitive decline and an elevated risk of dementia (Innes and Sambamoorthi, 2020); on the other, individuals in the early stages of neurodegeneration may experience heightened pain sensitivity beyond what can be explained by comorbidities alone (Cao et al., 2019; Marques and Brefel-Courbon, 2021). Mechanistically, the sustained neuronal activity, stress, and inflammation inherent to chronic pain can disrupt proteostasis (Brezic et al., 2025), manifesting as ER stress, impaired protein degradation, and accumulation of misfolded proteins, processes analogous to those observed in neurodegeneration (Deneubourg et al., 2022). The neuroinflammatory milieu characteristic of chronic pain may exacerbate oxidative and proteotoxic stress in neurons and glia, promoting pathological protein aggregation and thereby initiating neurodegenerative changes (Reichling and Levine, 2011; Tian et al., 2024). Consequently, a paradigm shift is emerging: chronic pain should no longer be viewed merely as a consequence of certain neurological disorders, but rather as an active contributor to neurodegenerative processes through shared pathological mechanisms (Brezic et al., 2025). The present study demonstrates that, under NP conditions, DEGs in insular subregions are enriched in pathways associated with neuroinflammation, ER and mitochondrial function, and calcium signaling. These findings provide cortical-level evidence supporting the proposed mechanistic overlap between chronic pain and neurodegeneration, offering new insights into the NP pathophysiology.

### 4.3 Other pathways of neuropathic pain in the insular cortex

In this study, KEGG pathway analysis also revealed involvement of the PI3K/Akt pathway and circadian rhythm regulation within the IC under NP conditions. The PI3K/Akt pathway was enriched in the aIC at both 2 and 4 weeks after SNI. In contrast, circadian rhythm-related pathways were enriched in both the aIC and pIC only at the 4-week time point. PI3K functions as a dual-specificity enzyme with both protein and lipid kinase activities. Upon activation by cytokines and growth factors, PI3K promotes Akt phosphorylation, which in turn modulates downstream effectors involved in cell survival, proliferation, and metabolism (Vilar et al., 2011). Despite its well-characterized roles in these fundamental processes, evidence implicating PI3K/Akt signaling in cortical pain mechanisms remains sparse. To date, only one study has reported that pharmacological inhibition of this pathway in the ACC alleviates both sensory and affective pain behaviors in a rodent model of cancer-induced pain (Liu et al., 2025), highlighting a critical gap in our understanding of how this signaling cascade contributes to NP at the cortical level.

Clinical evidence has firmly established that NP is subject to circadian modulation (Burish et al., 2019). To date, mechanistic investigations have primarily focused on peripheral and spinal sites, as well as descending pain modulatory pathways (Bumgarner et al., 2021). By contrast, direct evidence implicating higher-order cortical and limbic structures in the circadian gating of pain perception remains remarkably limited. Nonetheless, molecular oscillations with clear diurnal rhythms have been documented within these brain regions (Logan et al., 2022). Extending these observations, our findings reveal a previously underappreciated role for circadian rhythm disruption within the IC in NP pathogenesis. Future work will be essential to delineate the precise mechanisms by which cortical and limbic circuits contribute to the circadian organization of pain processing.

### 4.4 Region-specific transcriptomic remodeling of the insular cortex in neuropathic pain

As shown in Figure 5, SNI induced region-specific and time-dependent transcriptomic remodeling in the IC. The aIC exhibited broader and more persistent pathway alterations than the pIC, including programmed cell death (early time point) and oxidative stress–mitochondrial dysfunction–neurodegeneration (late time point), indicating a progressive stress response unique to the aIC. In contrast, both subregions retained core synaptic and neuroinflammatory pathways, suggesting a conserved functional reprogramming across the IC in chronic pain. These findings provide important clues for understanding the divergent molecular mechanisms of insular subregions in neuropathic pain.

The aIC and pIC differ markedly in histological architecture (Gogolla, 2017), fiber connectivity (Gehrlach et al., 2020) and electrophysiological properties (Iezzi et al., 2024), which may underlie their differential vulnerability to pain induced transcriptomic dysregulation. In terms of thalamic connectivity, both subregions receive inputs from the ventral posteromedial (VPM) and posterior (Po) nuclei, but the aIC additionally receives projections from the mediodorsal (MD) nucleus and other higher order thalamic nuclei (Jasmin et al., 2004; Gehrlach et al., 2020). Consequently, the pIC primarily processes sensory discriminative aspects of pain, whereas the aIC additionally integrates affective motivational signals (Lu et al., 2016). This higher order integrative function probably imposes greater and more sustained regulatory demands on aIC neurons under chronic pain conditions, leading to protracted activation of synaptic plasticity pathways, accumulation of oxidative damage and eventual engagement of cell death programs—a pattern not observed in the pIC. Thus, the heightened susceptibility of the aIC to pain related molecular pathology may be an inherent consequence of its core function as an affective motivational hub. Beyond fiber connectivity, local molecular differences between aIC and pIC, such as receptor and transmitter systems, may also contribute to their divergent transcriptomic profiles, a possibility that warrants future investigation using single cell transcriptomic, proteomic and metabolomic approaches.

### 4.5 Cross-dataset validation strengthens the robustness of key transcriptomic signatures

A potential limitation of single-dataset transcriptomic studies is the risk of false positives from batch effects, sample heterogeneity, or analytical variability. Here, we performed an independent RNA-seq analysis on the cingulate cortex from the same neuropathic pain cohort. This internal cross-validation design allowed us to assess whether key DEGs and pathways identified in the IC could be independently recapitulated in the cingulate cortex, another pain-related cortical region. Several lines of evidence support our findings. First, DEG temporal trajectories in the cingulate cortex mirrored those in the IC—both showed increased DEG numbers from 2 to 4 weeks after SNI, indicating progressive transcriptional reprogramming during chronic pain maintenance. Second, at 4 weeks post-SNI, overlapping DEGs between the aIC and ACC were significantly enriched in mitochondrial dysfunction and neurodegenerative disease pathways, also observed in the anterior insular cortex-specific analysis. This cross-regional convergence suggests chronic pain induces a conserved, anterior-subregion-preferring signature involving oxidative stress and impaired energy metabolism. Third, pathways related to MAPK signaling, neuroactive ligand-receptor interaction, protein digestion and absorption, and circadian rhythm were significantly enriched across all overlapping datasets, confirming these processes represent fundamental transcriptional responses to persistent pain across higher-order association cortices. These results reinforce the proposed mechanistic link between chronic pain and neurodegenerative disorders at the cortical level. Future studies integrating single-cell and spatial transcriptomics across multiple cortical regions are needed to determine whether these shared signatures arise from common cell-type-specific programs.

### 4.6 Limitations

Several limitations should be acknowledged in the present study. First, the IC comprises heterogeneous cell populations, and our analyses were derived from bulk tissue, which integrates signals across these diverse cell types. Ongoing snRNA-seq and spatial transcriptomics studies in our laboratory will resolve cell-type-specific and spatially localized transcriptional changes in the IC under NP conditions, extending the findings reported here. Second, while our study focused on transcriptional and pathway level alterations, it did not assess post-translational modifications—particularly rapid, phosphorylation dependent regulatory mechanisms known to modulate higher brain functions. Integrating multi-omics approaches, such as phosphoproteomics, metabolomics, and lipidomics, could reveal more refined molecular distinctions underlying NP. Third, while we identified transcriptomic changes in the IC, their causal role in NP initiation or persistence, and their therapeutic potential, require experimental validation. Further mechanistic and interventional studies are needed to confirm functional relevance and translational value. Fourth, advanced machine learning methods such as Random Forest or Least Absolute Shrinkage and Selection Operator regression (LASSO) regression could theoretically identify key genes more robustly (Reel et al., 2021), and future application of these approaches is necessary to cross-validate our current findings, thereby enhancing the depth and robustness of the analyses.

## 5 Conclusion

Collectively, these results provide compelling evidence that neuroinflammation and synapse-related mechanisms within the IC contribute to the pathophysiology of NP. Notably, our findings reveal region- and time-dependent transcriptomic remodeling: the aIC exhibits broader and more persistent alterations than the pIC, including a progressive stress response that recapitulates features observed in neurodegenerative disorders, indicating its heightened susceptibility to pain*induced pathology. By elucidating these region-specific and time-dependent molecular signatures, our study enhances the mechanistic understanding of NP and establishes a conceptual framework for the development of novel therapeutic strategies for chronic pain.

## Data Availability

The datasets presented in this study can be found in online repositories. The names of the repository/repositories and accession number(s) can be found in the article/Supplementary material.

## Supplementary material

Supplementary Figure 1 Quality assessment of the RNA-sequencing dataset across all samples.

Supplementary Figure 2 Cell-type enrichment of DEGs induced in insular subregions after SNI.

Supplementary Figure 3 Construction of the weighted gene co-expression network.
