# Supplementary material for: Transcriptomic signatures of the insular cortex in a mouse model of neuropathic pain
Source: Front Mol Neurosci. 2026 Jul 2;19:1840950. doi: 10.3389/fnmol.2026.1840950 (PMC13372706; doi:10.3389/fnmol.2026.1840950)
Supplement: Supplementary file 1 [file Supplementary_file_1.docx]

**Supplemental Table 1.** Summary of RNA-sequencing data quality metrics.

| Sample | Raw data read | Valid data read | Valid ratio% | Mapped rate% | Q20% | Q30% |
| --- | --- | --- | --- | --- | --- | --- |
| Sham aIC1 | 22159080 | 21981077 | 99.20 | 0.9870 | 98.97 | 95.31 |
| Sham aIC2 | 23180410 | 22987993 | 99.17 | 0.9860 | 98.96 | 95.29 |
| Sham aIC3 | 20977869 | 20812900 | 99.21 | 0.9850 | 98.99 | 95.38 |
| Sham aIC4 | 25683095 | 25471325 | 99.18 | 0.9870 | 98.95 | 95.25 |
| Sham aIC5 | 26771567 | 26559505 | 99.21 | 0.9880 | 99.00 | 95.45 |
| Sham aIC6 | 20941187 | 20762564 | 99.15 | 0.9880 | 98.95 | 95.25 |
| SNI-2w aIC1 | 26266110 | 26053376 | 99.19 | 0.9880 | 98.99 | 95.43 |
| SNI-2w aIC2 | 25625166 | 25420744 | 99.20 | 0.9880 | 99.03 | 95.57 |
| SNI-2w aIC3 | 23123003 | 22951902 | 99.26 | 0.9880 | 98.96 | 95.20 |
| SNI-2w aIC4 | 25957437 | 25749111 | 99.20 | 0.9860 | 98.94 | 95.20 |
| SNI-2w aIC5 | 27233912 | 27021342 | 99.22 | 0.9880 | 98.98 | 95.36 |
| SNI-2w aIC6 | 23869644 | 23681360 | 99.21 | 0.9870 | 98.94 | 95.22 |
| SNI-4w aIC1 | 23215543 | 23021217 | 99.16 | 0.9870 | 98.95 | 95.26 |
| SNI-4w aIC2 | 26265948 | 26063763 | 99.23 | 0.9880 | 99.03 | 95.50 |
| SNI-4w aIC3 | 22656865 | 22480508 | 99.22 | 0.9860 | 98.93 | 95.09 |
| SNI-4w aIC4 | 24857048 | 24655409 | 99.19 | 0.9870 | 98.90 | 94.99 |
| SNI-4w aIC5 | 25913051 | 25699926 | 99.18 | 0.9870 | 99.01 | 95.51 |
| SNI-4w aIC6 | 21465523 | 21291646 | 99.19 | 0.9880 | 98.97 | 95.34 |
| Sham pIC1 | 23262375 | 23077573 | 99.21 | 0.9880 | 98.96 | 95.25 |
| Sham pIC2 | 26571451 | 26342471 | 99.14 | 0.9860 | 98.98 | 95.42 |
| Sham pIC3 | 25723595 | 25503169 | 99.14 | 0.9870 | 98.97 | 95.34 |
| Sham pIC4 | 21827390 | 21669753 | 99.28 | 0.9880 | 99.04 | 95.54 |
| Sham pIC5 | 22168418 | 21971078 | 99.11 | 0.9870 | 98.96 | 95.34 |
| Sham pIC6 | 23287868 | 23094725 | 99.17 | 0.9870 | 98.90 | 94.96 |
| SNI-2w pIC1 | 26041326 | 25826969 | 99.18 | 0.9880 | 98.98 | 95.38 |
| SNI-2w pIC2 | 27042748 | 26824912 | 99.19 | 0.9880 | 99.00 | 95.41 |
| SNI-2w pIC3 | 22658730 | 22476617 | 99.20 | 0.9890 | 99.04 | 95.61 |
| SNI-2w pIC4 | 26518969 | 26311560 | 99.22 | 0.9890 | 99.07 | 95.74 |
| SNI-2w pIC5 | 24542623 | 24341597 | 99.18 | 0.9890 | 98.94 | 95.09 |
| SNI-2w pIC6 | 26334233 | 26100825 | 99.11 | 0.9870 | 98.95 | 95.30 |
| SNI-4w pIC1 | 23194869 | 23005775 | 99.18 | 0.9870 | 98.95 | 95.25 |
| SNI-4w pIC2 | 22830604 | 22635976 | 99.15 | 0.9870 | 98.96 | 95.27 |
| SNI-4w pIC3 | 23545706 | 23337022 | 99.11 | 0.9880 | 98.99 | 95.42 |
| SNI-4w pIC4 | 22356258 | 22205340 | 99.32 | 0.9880 | 99.03 | 95.46 |
| SNI-4w pIC5 | 22504597 | 22339314 | 99.27 | 0.9870 | 99.07 | 95.71 |
| SNI-4w pIC6 | 23837030 | 23667909 | 99.29 | 0.9880 | 99.03 | 95.52 |

This table provides a comprehensive overview of 36 individual samples, including the number of raw and clean reads, the valid ratio, the percentage of successfully mapped reads, and the base-calling quality scores (Q20 and Q30).

**Supplemental Table 2.** A reference panel comprising genes encoding synapse-related molecules, G protein-coupled receptors, ion channels, and neuropeptides.

| Types | Genes |
| --- | --- |
| Synapse-related molecules | Agt Cadps2 Chat Cplx1 Napb Slc17a7 Slc18a3 Slc5a7 Snca Sncg Stxbp5l Sv2b Syt6 Syt9 Ywhaz Abat Atp2a2 Atp2b2 Baiap3 Cadps Camk2a Cd47 Clu Lin7a Nat8l Nlgn1 Nrxn3 Ntrk2 Pak1 Pde1b Rph3a Sirpa Slc17a6 Slc1a2 Slc6a1 Slc6a7 Sv2a Syn2 Syp Syt11 Vamp1 Ryr2 Ryr3 Itpr1 Itpr2 Itpr3 Nlgn1 Pde4b Calm1 |
| Ion channels | Ano1 Cacnb3 Cacng5 Chrna3 Chrna4 Chrnb3 Chrnb4 Kcna2 Kcnd2 Kcng4 Kcnip1 Kcnip3 Kcnma1 Kcnmb4 Lrrc55 Cacna1b Cacna1g Cacna1i Cacna2d1 Cacng2 Gabrb1 Gabrg2 Gabrg3 Glrb Hcn2 Kcna6 Kcnab2 Kcnc1 Kcnc2 Kcnc4 Kcnip4 Kcnj3 Scn1a Scn1b Ttyh1 Gabra1 Gabra2 Gabra3 Gabra4 Gabra5 Gabrb1 Gabrb2 Gabrb3 Gria1 Gria2 Gria3 Gria4 Grid1 Grid2 Grik1 Grik2 Grik3 Grik4 Grik5 Grin1 Grin2a Grin2b Grin2c Grin3a P2rx1 P2rx4 P2rx7 |
| G protein-coupled receptors | Ackr1 Ackr3 Adcyap1r1 Adgra1 Adgra2 Adgra3 Adgrb1 Adgrb2 Adgrb3 Adgrd1 Adgre1 Adgre5 Adgrf4 Adgrf5 Adgrg1 Adgrg2 Adgrg3 Adgrg6 Adgrl1 Adgrl2 Adgrl3 Adgrl4 Adgrv1 Adora1 Adora2b Adora3 Adra1a Adra1b Adra2a Adra2b Adra2c Adrb1 Adrb2 Adrb3 Agtr2 Agtrap ApInr Avpr1a C3ar1 C5ar1 C5ar2 Calcrl Cckbr Ccr1 Ccr10 Ccr5 Ccr7 Ccrl2 Celsr1 Celsr2 Celsr3 Chrm1 Chrm2 Chrm3 Chrm5 Cmklr1 Cnr1 Crcp Crhr1 Crhr2 Cx3cr1 Cxcr2 Cxcr4 Cysltr1 Drd1 Drd4 Drd5 Ednra Ednrb F2r F2rl3 Ffar1 Fpr2 Fzd1 Fzd10 Fzd2 Fzd3 Fzd4 Fzd5 Fzd6 Fzd7 Fzd8 Fzd9 Gabbr1 Gabbr2 Galr1 Ghsr Gipr Gper1 Gpr1 Gpr101 Gpr12 Gpr135 Gpr139 Gpr141 Gpr149 Gpr150 Gpr151 Gpr152 Gpr153 Gpr156 Gpr157 Gpr158 Gpr160 Gpr161 Gpr162 Gpr17 Gpr173 Gpr176 Gpr182 Gpr19 Gpr21 Gpr22 Gpr26 Gpr27 Gpr3 Gpr34 Gpr35 Gpr37 Gpr37l1 Gpr4 Gpr45 Gpr50 Gpr55 Gpr6 Gpr61 Gpr62 Gpr68 Gpr75 Gpr83 Gpr85 Gpr88 Gprc5b Gprc5c Grm1 Grm2 Grm3 Grm4 Grm5 Grm7 Grm8 Grpr Hcrtr1 Hcrtr2 Hrh1 Hrh2 Hrh3 Htr1a Htr1b Htr1d Htr1f Htr2a Htr2c Htr4 Htr5a Htr5b Htr6 Htr7 Kiss1r Lgr4 Lgr5 Lgr6 Lpar1 Lpar4 Lpar6 Ltb4r1 Mc1r Mc3r Mc4r Mchr1 Mtnr1a Nmbr Nmur2 Npbwr1 Npr3 Npy1r Npy2r Npy5r Ntsr1 Ntsr2 Ogfr Ogfrl1 Oprk1 Oprl1 Oprm1 Oxgr1 Oxtr P2ry1 P2ry12 P2ry13 P2ry14 P2ry2 P2ry6 Pgr15l Ppard Prokr2 Ptgdr Ptger1 Ptger2 Ptger3 Ptger4 Ptgfr Ptgir Pth1r Ramp1 Ramp2 Ramp3 Rorb Rxfp1 Rxfp2 Rxfp3 S1pr1 S1pr2 S1pr3 S1pr5 Sigmar1 Smo Sstr1 Sstr2 Sstr3 Sstr4 Sstr5 Sucnr1 Tacr1 Tacr3 Tas1r1 Tas1r3 Tbxa2r Trhr Trhr2 Uts2r Vipr1 Vipr2 Vmn1r4 Vmn2r29 Vmn2r84 Vmn2r87 Xcr1 |
| Neuropeptides | Adcyap1 Agrp Calca Cartpt Cort Gal Grp Hcrt Nmb Nms Npb Npff Nppa Nps Npy Pcsk1n Pdyn Penk Pnoc Pomc Prok2 Pth2 Qrfp Scg5 Tac1 Tac2 Ecel1 Tenm1 Tyro3 Rapgef2 Sort1 |
