# Supplementary figures and images for: Transcriptomic signatures of the insular cortex in a mouse model of neuropathic pain
Source: Front Mol Neurosci. 2026 Jul 2;19:1840950. doi: 10.3389/fnmol.2026.1840950 (PMC13372706; doi:10.3389/fnmol.2026.1840950)

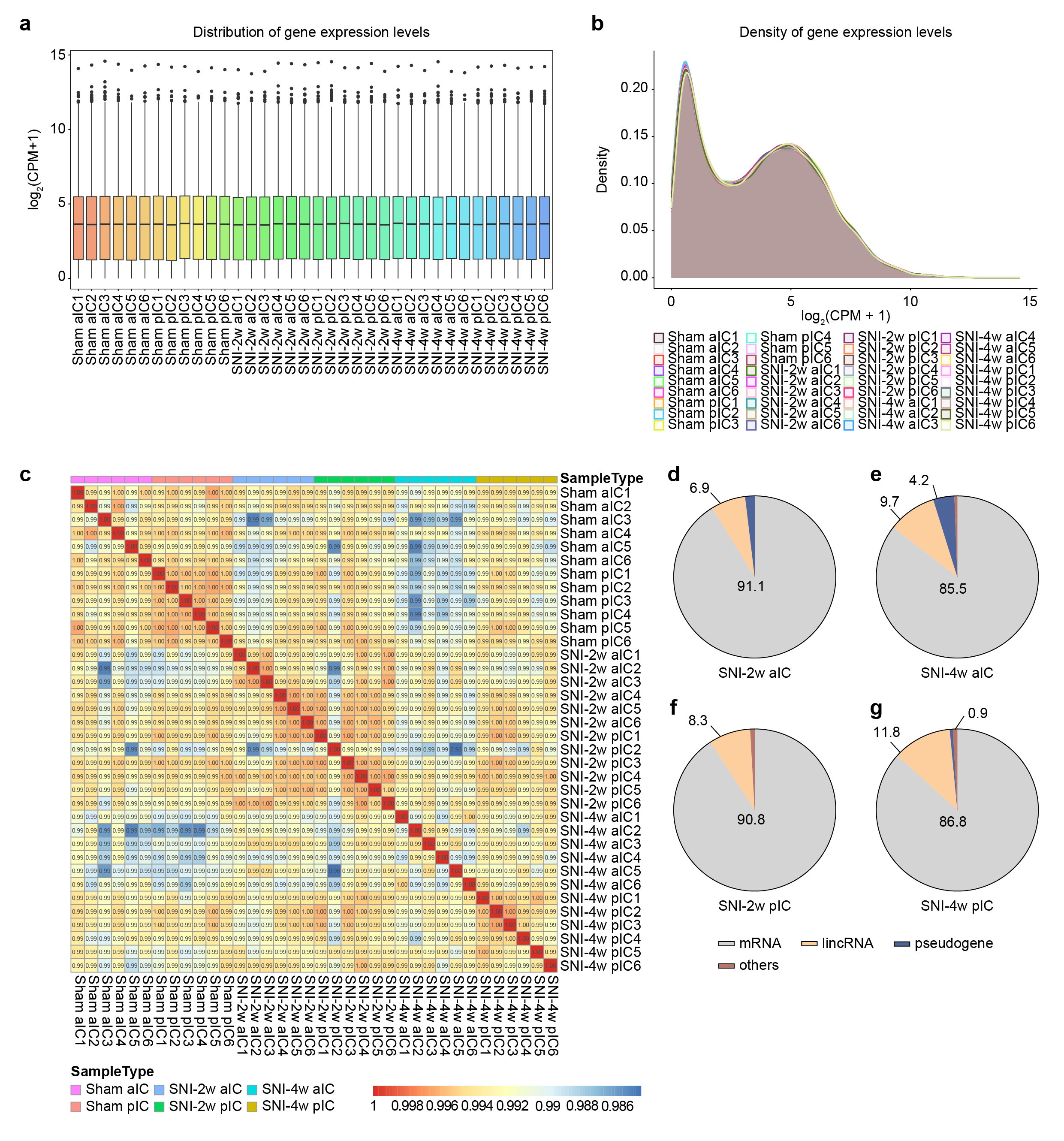

Supplement: Supplementary Figure 1 — Quality assessment of the RNA-sequencing dataset across all samples. [file Image_1.tiff]

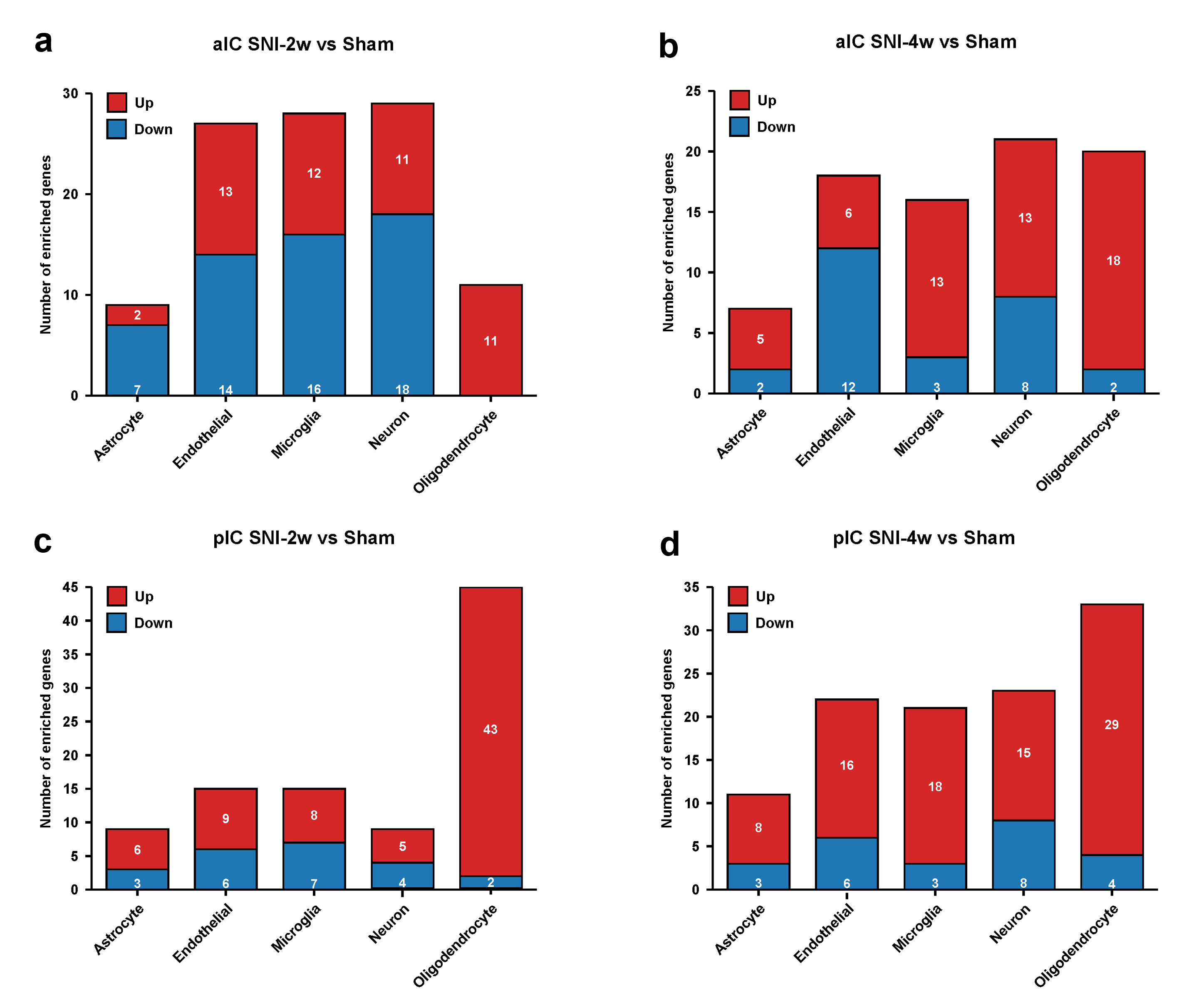

Supplement: Supplementary Figure 2 — Cell-type enrichment of DEGs induced in insular subregions after SNI. [file Image_2.tif]

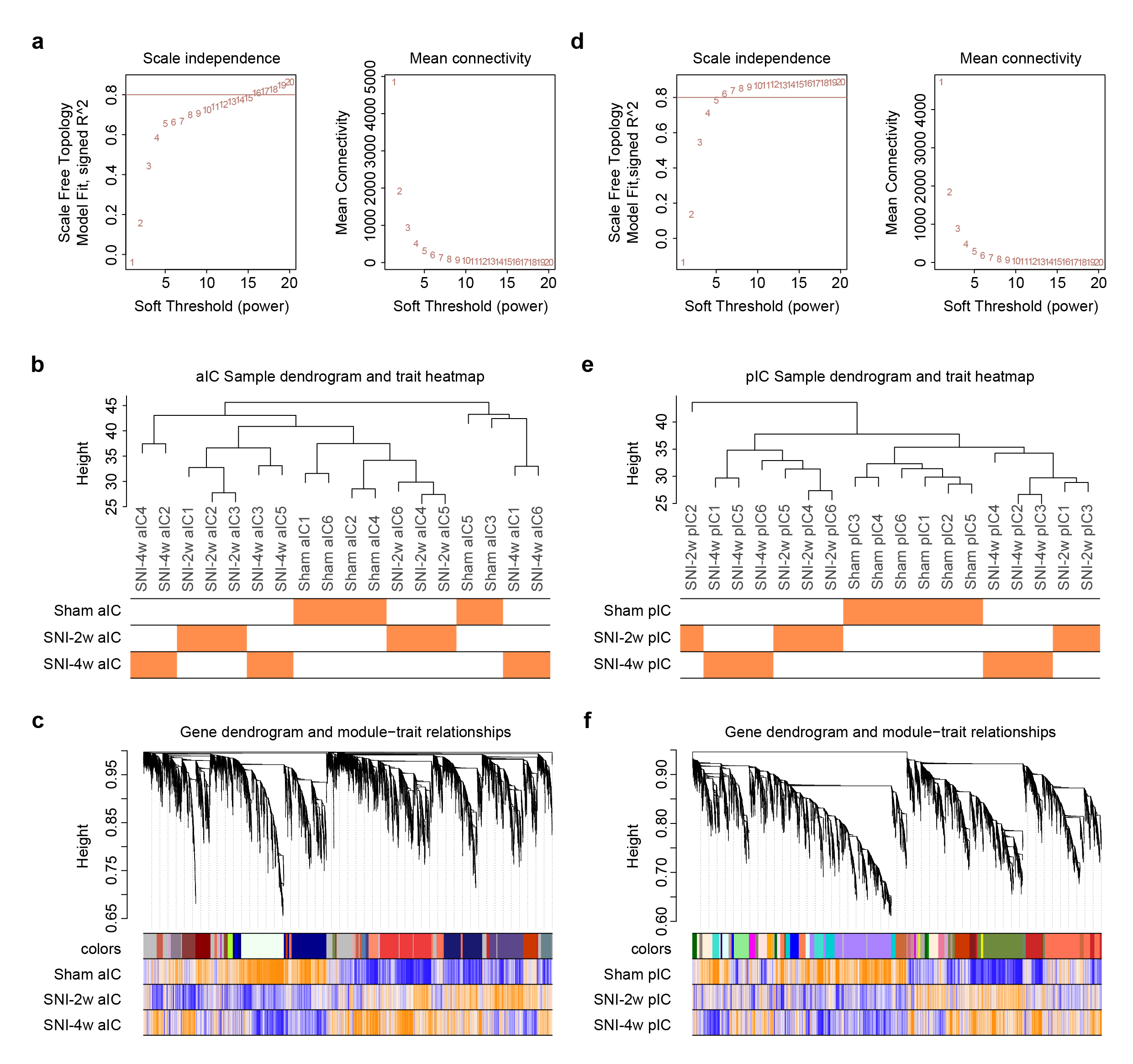

Supplement: Supplementary Figure 3 — Construction of the weighted gene co-expression network. [file Image_3.tiff]
